# Synthesis and applications of novel Schiff base derivatives as corrosion inhibitors and additives for improvement of reinforced concrete

**DOI:** 10.1038/s41598-023-41165-7

**Published:** 2023-09-12

**Authors:** Ehab S. Gad, Mohamed A. Abbas, Mahmoud A. Bedair, Olfat E. El-Azabawy, Shymaa M. Mukhtar

**Affiliations:** 1 Chemistry Department, College of Science and Arts, Jouf University, Alqurayat, Saudi Arabia; 2 Egyptian Petroleum Research Institute (EPRI), Cairo, 11727 Egypt; 3 Department of Chemistry College of Science and Arts, University of Bisha, P.O. Box 101, 61977 Al-Namas, Saudi Arabia; 4 Civil Engineering Department, Higher Technological Institute, 10th of Ramadan City, Sharqeya Egypt

**Keywords:** Engineering, Materials science

## Abstract

The studied Schiff-base compounds in this work are multitasked investigated as corrosion inhibitors and also, to improve the physical and mechanical properties of reinforced concrete. The efficiency inhibition of the two novel Schiff-base compounds named (DHSiMF) and (DHSiB) for corrosion of carbon-steel in aqueous media of 1 M HCl was assessed via electrochemical methods and loss in weight. FT-IR, ^1^H-NMR spectra and elemental analysis were used to confirm the structure of such compounds. It was found to have successful inhibition even at low concentrations in tested media, as an increase in inhibitor concentration led to an improvement in the inhibition efficiency. The weight loss results clearly demonstrate that DHSiMF of C-steel in 1 M HCl has a higher inhibition efficiency than DHSiB, with a maximum inhibition efficiency (85%) attained at 1 × 10^–2^ M from DHSiMF. Electrochemical experiments likewise revealed the same order, but with a maximal inhibitory efficiency of 98.1%. The addition of inhibitors to the corrosive media dramatically changed the anodic Tafel constants (β_a_) and cathodic Tafel constants (β_c_), indicating a mixed type nature. Electrochemical polarization curves illustrated the functions of mixed-type inhibition and the action of adsorption matching with the Langmuir adsorption isotherm. The ∆G_ads_ values for DHSiMF and DHSiB at temperatures (ranging from 303 to 333 K) are − 34.42 kilojoule/mole to − 37.51 kilojoule/mole. These values indicate that the compounds’ adsorption types are chemo-physical adsorption. X-ray diffraction (XRD) and Scanning electron microscopy (SEM) experiments were used to check the existence of the protection layer on the surface of carbon steel by analyzing the morphologies of the corrosion effects and the formed chemical compositions of the corrosion outcomes. For the concrete, the findings suggest that the chemical reaction that takes place between the DHSiMF and DHSiB and the concrete mix will result in an increase in the flexural strength, the compressive strength, and the indirect tensile strength of the concrete that is made of the gravel and dolomite aggregate.

## Introduction

Corrosion attack is a global issue in industrial applications as it disturbs emerging and developed countries’ economies^1–4^. It is possible to think of it as a chemical process that converts the surfaces of metals into the proper ionic species in the presence of moisture (H_2_O) and other reactive chemicals in the immediate area. Protecting machinery that is prone to corrosion can be accomplished by utilizing items such as hydrochloric acid or sulfuric acid for the removal of mill scale, acid washing, well-acidifying oil, and the removal of localized deposits after processes have been halted. Carbon steel alloys are usually agreed to start with HCl as a media^5,6^. Creating a separating layer between the metal and the surrounding medium is the main objective for which many researchers have made efforts to prevent the oxidation of the steel layer under different conditions. Recently, the utilization of inhibitors has been applied in the most appropriate processes of preventing corrosion in steel. Organic inhibitors have a heteroatom such as nitrogen, sulfur, and oxygen are the more active commercial ones^7,8^. The less electronegative heteroatom compared with the relatively more electronegative heteroatom has stronger metal-inhibitor interactions^9^. The compounds of steel corrosion inhibitors in aggressive media are aliphatic, aromatic amines and heterocyclic of nitrogen compounds. The adsorption function of bio-inhibitors on the surfaces is to disrupt active positions by expelling the molecules of water adjacent to the surface of metal forming a condensed layer film to lower the corrosion rate^10^. The handle of adsorption and the impediment component rely on electronic features of inhibitor, design of surface, corrosion-related conditions and the components of aggressive media^11^. Various techniques, (potential-dynamic polarization, electro-chemical impedance spectroscopy, loss in weight, etc.), are used to assess the effectiveness of the inhibitor in such studies. There have been many researchers have merged these methods^12,13^. The first records of this form of reaction were published in 1860^14,15^. Numerous inhibitors of Schiff-bases have been produced for aqueous media for a variety of metals and alloys^16^. The compound contains an azomethine group (–CHN–), therefore the Schiff-bases will function as efficient inhibitors. The substitution of other elements also affects the activity of inhibition, in addition to the group of imines^17^. It has been established that certain Schiff base compounds show greater inhibition activity than their related amine and carbonyl compound^18^. Both the early (7th day) and standard (28th day) curing times were used to test the mechanical strength of the samples.in these studies of the effect of Nano silica and silica fume on the structural features of cement mortars^19^. At both ages, it was discovered that the mortar specimens containing NS particles had significantly better compressive and flexural strengths than the comparable specimens of SF over and above the control^20^. These gains in mortar strength are a result of NS large surface area and nano-sized particles. As nucleating agents, nanoparticles promoted the hydration of C3S and C2S and the formation of the C–S–H phase. The quantity of active sites on the surface of NS particles boosted their pozzolanic reactivity and the strength of the connections formed with free CH^21^. Around the world, concrete is often made with extra cementitious materials (SCMs) like silica fume (SF), fly ash (FA), and rice husk ash (RHA)^22,23^. In the presence of water, calcium hydroxide and silica fume react to form calcium silicate hydrate-based cementitious compounds. The addition of silica fume to concrete improves its strength and durability. In addition, silica fume has apparently been employed well to produce chemically resistant concrete with extraordinarily high strength and low permeability. A research found, however, that employing 10% silica fume as a cement replacement ingredient while producing 70-MPa concrete might have a positive influence on corrosion rate^24,25^. The purpose of this study is to investigate the effectiveness of new Schiff base compounds (DHSiMF and DHSiB) in inhibiting the corrosion of carbon steel in a solution of 1 M hydrochloric acid, both in the absence of these compounds and in their presence, using weight loss and electro-chemical measurements. The effectiveness of the inhibitors DHSiMF and DHSiB in preventing effects has also been evaluated through the use of a weight loss procedure at a range of temperatures. Methods like as scanning electron microscopy (SEM) and x-ray powder diffraction were also utilized in order to evaluate the morphological characteristics of the corrosion products that were produced. Concrete is one of the most important an unsustainable material that must be studied and searched for different ways to improve and develop its physical and mechanical properties because it is an essential and important entity in all fields. There are many companies producing concrete additives to improve its properties, but many of these additives are harmful to the environment, so it is necessary to search for other environmentally friendly materials and this is the main reason for using organic-silicone compounds.

## Materials and methods

### Steel composition and solutions

The main element of carbon steel is Fe (99.0483%) in addition of other minor elements C (0.09073%), Si (0.04448%), Mn (0.48640%), P (0.01246%), S (0.02186%), Ni (0.05200%), Cr (0.04218%), W (0.00754%), Mo (0.01744%) and Cu (0.14905%). By combining bi-distilled water with AR class 37% HCl, the violent 1.0 M HCl solutions are produced. For both DHSiMF and DHSiB, their concentration range is between 5 × 10^–6^ and 1 × 10^–2^ M/L. All solutions were made with bi-distilled water.

### Synthesis pathway of the investigated Schiff bases

**Figure 1:**
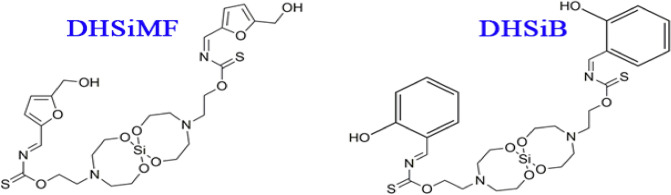
The chemical formula of the prepared Schiff base compounds.

1 Mole of Bis (Dithio-amine Triethanol-amine) Siloxane^26^ with 2 mol either 5-(Hydroxy-methyl) furan-2-carbaldehyde or 2-Hydroxy benzaldehyde were put into a 3-necked round bottom flask, heat to 120 °C and hold for 2 h to produce new compounds under named Di[O-(5-(hydroxymethyl)furan-2-yl)-methylenemethanethioamide)-tetraoxa-diaza-silaspiropentadecan-4-yl)ethyl) (DHSiMF) and Di[O-2-hydroxybenzylidene) carbamothioyl) oxy)ethyl)-1,7,9,15-tetraoxa-4,12-diaza-8-silaspiro[7.7]pentadecan-4-yl)ethyl)((Z)-2-hydroxybenzylidene) carbamothioate (DHSiB). The flask was hooked up to a device for distillation at 250 °C, and it was stirred constantly for 2 h. On cooling to the room temperature, the reaction mixture was isolated by filtration. The chemical structures of the prepared Schiff bases are shown in Fig. 1.

Elemental scheme of FT-IR and ^1^HNMR spectra confirmed the chemical characteristics of the prepared compound. Elementary compositions are performed using Vario Elementar instrument for the prepared samples. In the range of 500–4000 cm^–1^, a Mattson Bench Top 961 FT-IR spectrometer was employed. The different bands of various types of functional groups were recorded. ^1^HNMR spectrometer of 300 MHz, (W-P-300-Bruker), was used to report the prepared Schiff base silicon compounds in dimethyl sulphoxide.

### Weight loss measurements

Weight loss tests were done using rectangle specimens having 5.6 × 2.7 × 0.5 cm dimensions and surface area of 38.54 cm^2^. As the appropriate standard procedure, weight loss measurements were carried out by submerging the carbon steel coupons that had been produced in (1 M HCl) aerated offensive solutions. Temperatures between 303 and 333 K were used to investigate the effectiveness of inhibition with and without different amounts of produced Schiff base compounds. DHSiMF and DHSiB were dissolved directly in the solution being tested. Solutions were subsequently put into 150 mL sealed glass bottles and the coupons were suspended without stirring at room temperature in these solutions. After 24 h immersion time, the samples were collected, cleaned, dried with warm air of 40 °C and exactly weighed. The Eqs. (1), (2) estimated the surface coverage degree (Ө) and the efficiency of inhibition (η_w_ %) respectively^27^;

1 $$\theta ={W}_{o}-{W}_{inh.}/{W}_{o},$$

2 $${\eta }_{w}={W}_{o}-{W}_{inh.}/{W}_{o}\times 100,$$

where the values of weight loss given the symbol (${W}_{o}$ and ${W}_{inh.}$) without and with definite inhibitor concentrations individually. Equation (3) estimated the rate of corrosion C $R$ (mg cm^–2^ h^–1^)^28^.

3 $$CR=W/S*t,$$

where S and t are express the surface area of each tested specimen and the time of exposure respectively.

### Electrochemical measurements

For electrochemical tests, a cylinder of rebar with a surface area of 1 cm^2^ was coated with epoxy resin and then used as a working electrode. The electrode layer was abraded using a sequence of emery paper (320:1200) and cleaned with bi-distilled H_2_O, and acetone.

For polarization and impedance testing, a Volta Lab Master Radiometer (model PGZ 301) equipped with the Zsimpwin software application was used. Three electrode systems were deployed against a carbon steel working electrode, including a saturated calomel electrode reference system and a platinum wire counter electrode. To measure impedance, a 10 mV AC sine wave with an open circuit potential and a frequency range of 100 kHz to 50 mHz was used. For polarization measurements, a potential of (− 800:− 300 mV) was required at a scanning rate of 0.2 mVs^–1^. The electrochemical experiments were carried out at 30 °C (ASTM G3-74 and G-87). By extending the linear Tafel part of the anodic and cathodic curves to the corrosion potential axis, you can figure out how much corrosion is going on.

### Monte-Carlo (MC) and molecular dynamics (MD) simulations

The Fe (110) model was used to mimic the interaction of the two Schiff base molecules with the steel surface in the corrosive environments (1.0 M HCl) as it is more stable than the other (1 0 0) and (1 1 1) Fe planes^29,30^. Fe crystal split along (110) plane. The Fe (110) plane was expanded to 10 × 10 supercell contact using a vacuum slap of 30 Å at the top of the plane to achieve dimension of 22.341 × 22.341 × 48.422 Å^31^. The aqueous solution was introduced as 1 inhibitor molecule, 200 H_2_O, 20 H_3_O^+^ and 20 Cl^–^. MC and MD simulations were performed using the adsorption locator and Forcite Dynamics tools respectively implemented in BIOVIA Material Studio 2017 software^32^. Utilizing a Nose thermostat with a 298 K temperature setting, MD was performed. It is decided to use an NVT ensemble with a simulation duration of 50 ps and a time step of 1 fs. The force field used for the energy was COMPASS II.

### X-ray diffraction (XRD) measurements

It was utilized to describe the corrosive conditions of the metal and to identify the corrosion products that were spread throughout its surface. Used metal coupons were soaked in an offensive solution for 48 h, both with and without a properly determined concentration of DHSiMF and DHSiB. The coupons’ rust was eliminated, finely ground, and then homogenized. The phases were identified using X-ray powder diffractometry (X'PERT-PRO-MPD-PANalytical, Netherlands). At 40 kV/40 mA and 1.5406 A^o^, the resultant patterns for Cu K radiation were obtained. The samples were examined incrementally in the range of 4°–80° with 0.02-degree step sizes and a phase time 0.40-s phase period.

### Scanning electron microscope (SEM) measurements

It was used to collect data on changes to the corrosive sample layer. The coupons were immersed in a 1 M HCl solution, separated, and washed with bi-distilled water/acetone before being dried in cold air at room temperature for 48 h in the presence or absence of the investigated Schiff base. In contrast, SEM examination is performed on the hydrated cement paste derived from the powder samples. With a resolution of × 5000, the SEM analysis scale was 5 m with a range of 5 m. Using image analysis, the quantity and distribution of fractures in concrete under various loading conditions were analyzed. The photos were captured using a JEOL JSM-53000 SEM.

### Aggregate types

On the other hand, a total of 180 concrete specimens were cast and tested in this research, divided into two groups G1, G2 with dolomite and gravel respectively as a coarse aggregate. Each group contains 36 cubes with dimensions 150*150*150 mm, 36 cylinders with 150 mm diameter and 300 mm height, and 18 beams with cross section 100*100 mm and length 500 m. These specimens were classified into six groups according to the concentration of siloxane dosages by mixing water. Only the first control group (A) without siloxane addition and the other five groups (B, C, D, E, F) containing siloxane with concentration 100, 200, 300, 400, 500 ppm receptivity in mixing water.

Aggregate consisted of fine and coarse aggregate. Harsh desert very fine sand was used as a fine aggregate with fineness modulus equal 2.243, it was almost free from impurities, silt, etc., Dolomite No 16 with S. G.2.5, and gravel with maximum nominal size 16 mm were used as coarse aggregate. For cement from El-Suez cement, in this study, conventional Portland cement of type CEM I 42.5N was employed. Testing of cement was carried out per the Egyptian Standard Specifications ESS-2421/2005. The drinking water was used for mixing, and the water-cement ratio was 0.6. Five concentrations of siloxane (100, 200, 300, 400, and 500 ppm) were added to the mixing water in order to form five mixes.

### Mix design and preparation of test specimens

On the other side, the mix design and preparation depended on the ACI Committee was used to compute the quantities of materials required for the test batch. Many trials mixes were made to adjust the proportions of the used materials to give the 250 kg/cm^2^ compressive strengths and suitable workability. The concrete mixes proportions required for one cubic meter of concrete are Cement 250 (kg), fine aggregate 670 (kg), coarse aggregate 890 (kg), water 150 (liter), and siloxane concentration 100, 200, 300, 400, and 500 ppm. A homogeneous concrete mixture was required, thus mixing procedures were carried out at a temperature of 20 to 25 °C. The specimens were de-molded and submerged in water for 7 and 28 days after being exposed for 24 h. At each of the test ages, each water-cured specimen was removed from the water, then it was rubbed with a clean and dry cloth to give it a saturated and dry surface.

### The compression tests

The compression test was carried-out on 72 cubes specimens in which three specimens for each group of two ages at age of 7 and 28 days with using two kinds of coarse aggregate were tested and their average strength was taken into consideration.

On the other hand, the indirect tensile strength is the capacity of a material or structure to withstand indirect loads and the ultimate tensile strength of a material is calculated from the following Eq. (4):

4 $$Ft=2P/\pi .D.L.$$

### The indirect tension tests

The indirect tension test was carried-out on 72-cylinder specimens in which three specimens for each group of two ages at age of 7 and 28 days with using two kinds of coarse aggregate were tested and their average strength was taken into consideration. In the other side, the procedure for conducting a flexural strength test in compliance with the requirements was outlined.

### The flexural tests

The authors chose the four-point flexural test over the three-point test because it allows the specimens to experience a pure bending moment in the middle third of their length, as well as pure compressive and tensile stress in their cross-section due to the absence of shear force in that area. During a four-point flexural test, the cross-sections of the specimens are both compressed and stretched at the same time. The Flexural strength test was carried-out on 36 beam specimens 100*100*500 mm in which three specimens for each group at age of 28 days with using two kinds of coarse aggregate were tested and their average strength was taken into consideration due to following Eq. (5).

5 $$Ff=MY/I.$$

## Results and discussion

### Confirmation of chemical structure of the prepared Schiff bases

The accompanying OH stretching vibrations of the associated alcohols, phenolic benzene ring, and C–H furan ring, respectively, are responsible for the broad band seen in the FTIR spectra of (DHSiMF and DHSiB) in the 3348–3100 cm^–1^ range. The aliphatic C–H stretching vibration is what causes the two bands in the region of 2941 and 2887 cm^–1^. In addition to the C–H stretching vibration of the ethylene groups, the bands in the region of 1639 cm^–1^ that are indicative of C=N and may overlap and absorb in this range of 1700–1630 cm^–1^. The stretching vibration of the C_furan_=C_furan_ and the C_Aro_=C_Aro_ in the polar aliphatic methyl alcoholic type causes the 1620–1600 cm^–1^ and a shoulder at 1517 cm^–1^. The peak at 1372 cm^–1^ corresponds to CH_2_ and C–H groups bending, and the band at 1440 cm^–1^ is caused by in-plane deformation of –CH_2_ (CH_2_–C–N). The big peak at 1032 cm^–1^ caused by (C=S) and the absorption band at 1151 cm^−1^ are both related to ether vibrations (C–O–C). Finally, Si–OCH_2_ could be responsible for the band at 1071 cm^–1^. The majority of two novel compounds’ ^1^HNMR spectra are found at the triplet at δ1H 3.8 ppm, which is distinguished by the presence of methyl protons from H_2_C–O–C=S. The chemical shift of the methylene protons –CH_2_–(–CH_2_–N–C) correlates to the extremely strong triplet peak at δ1H 2.88 ppm (4H for methyl groups). The triplet peak at δ1H 2.60 ppm (8 H for CH_2_ methyl group in the side attached with tertiary amine and in the other side attached with methoxide group). On the other hand, the triplet peak appear at δ1H 3.89 is confirm the methyl group attach with (silaspiro [7.7] group). The signal at δ1H 7.95 and 8.27 ppm can be attributed to $\left(\mathrm{N}=\mathrm{C}-\mathrm{H}\right)\mathrm{for DHSiMF and DHSiB respectively}$ δ1H 4.39(4H for CH_2_ groups) attached with furan ring. The δ1H 5.12, and δ1H 11.11 ppm (2H for OH groups) ppm attributable to CH_2_–OH alcoholic and OH phenolic respectively. The heterocyclic and aromatic rings may have appeared at (6.42–6.92 and 6.93–7.65 ppm, respectively), according to the large range of ^1^H NMR chemical shift of the doublet attributed to furan protons. Elemental Analysis Calculated For C_26_H_36_N_4_O_10_S_2_Si: C, 47.55%; H, 5.50%; N, 8.53%; Si, 4.28, S, 9.76 and O, 24.36%; elemental analysis Found for C_26_H_36_N_4_O_10_S_2_Si: C, 48.10%; H, 6.79%; N, 9.39%; Si, 3.38, S, 9.11 and O, 23.23%, and Elemental Analysis Calculated For C_28_H_36_N_4_O_8_S_2_Si: C, 51.83%; H, 5.59%; N, 8.64%; Si, 4.33, S, 9.88 and O, 19.73%; elemental analysis Found for C_26_H_36_N_4_O_10_S_2_Si: C, 52.12%; H, 5.37%; N, 8.59%; Si, 4.23, S, 9.94 and O, 19.75%.

### Aggregate types

**Figure 2:**
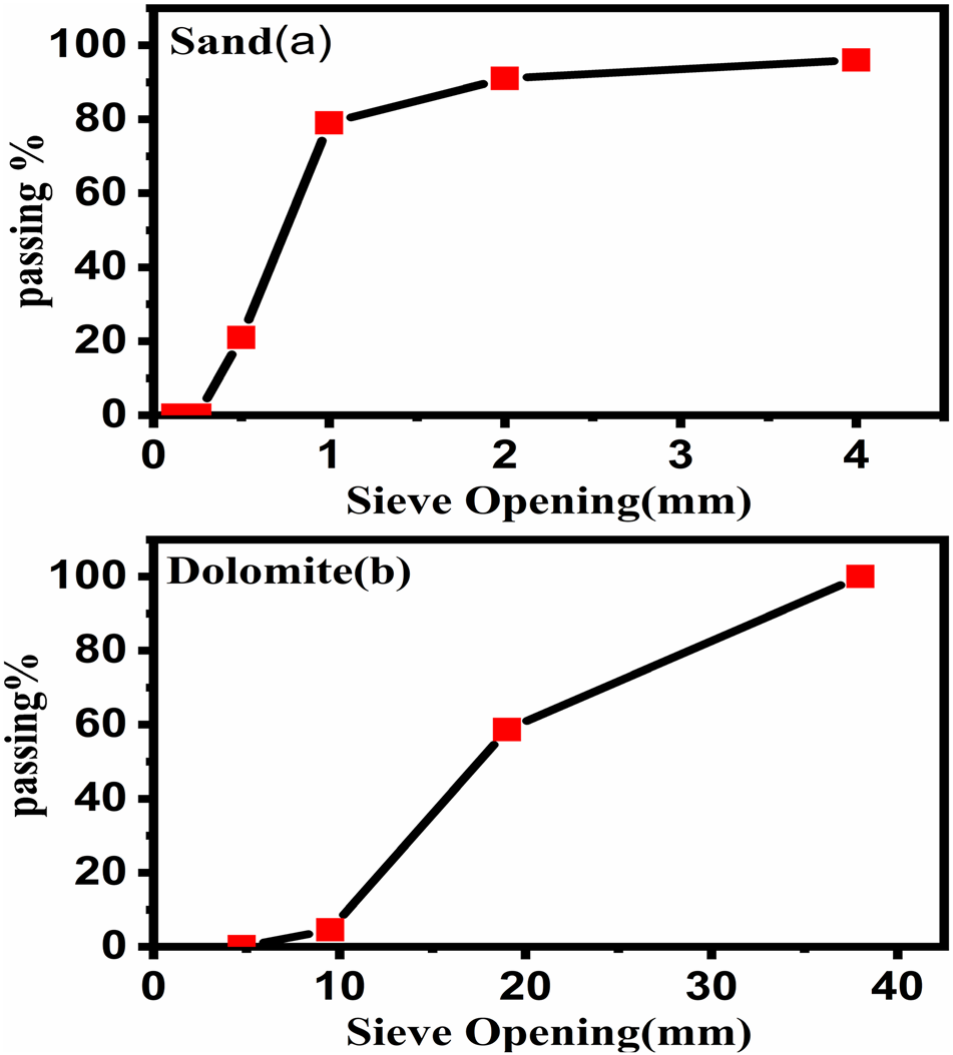
The diagram for the aggregate and sand grading used in the concrete mix.

Table 1 and Fig. 2 show that the physical properties for the aggregate and cement respectively. Bulk density or unit weight of an aggregate is the amount or weight required to fill a container to a specific unit volume, Mass/Volume equals bulk density^33^. Common building materials used to make normal-weight concrete have a bulk density of between 1200 and 1750 kg/m^3^.

**Table 1:** Physical and mechanical properties of the aggregate and cement.

Aggregate	Loose bulk density (B. D) (kg/m^3^)	Specific gravity (S.G.)	Finesse modulus	Presence of impurities and organic materials
Sand	1633.88	2.33	2.243	No
Gravel	1357.4	2.7	–	No
Dolomite	1541.66	2.5	–	No

Cement				
Property	Results	E.S.S limits		
Consistency of standard cement paste	Water content as percentage by weight of cement = 30%	26–33%		
Setting time	Initial = 90 min Final = 4 h., 30 min	Min. 45 min Max. 10 h		
Compressive strength	3 days = 25 N/mm^2^ 7 days = 34 N/mm^2^	Min. 18 N/mm^2^ Min. 27 N/mm^2^

From Table 1, the values of bulk density are 1634, 1357, and 1542 for sand, gravel, and dolomite respectively, these values for bulk density are agree with the value for the bulk density for common materials used in concrete. On the other side, the proportion of an aggregate’s mass to the mass of an equivalent volume of water is the relative density (specific gravity) of the substance^34^. The majority of aggregates have a relative density of 2.4 to 2.9 and a particle density of 2400 to 2900 kg/m^3^. The values for specific gravity computed in Table 1 are 2.33, 2.7, and 2.5 for sand, gravel, and dolomite respectively, these values are agreed with the standard values. On the other hand, the cement properties (Consistency of standard cement paste, Table 1 calculates setting time and compressive strength, these values for cement properties are agreement with the ESS limits. Figure 2 shows the plot between the particle size for sand, gravel, and dolomite with the sieve opening (mm). The particle size for dolomite is 0, 4.55, 58.7 and 100 with the sieve opening is 4.75, 9.5, 19 and 38 respectively. The particle size for sand is 0, 0, 21, 79, 91 and 96 with the sieve opening is 0.13, 0.25, 0.5, 1, 2 and 4 respectively.

### Open circuit potential measurements

**Figure 3:**
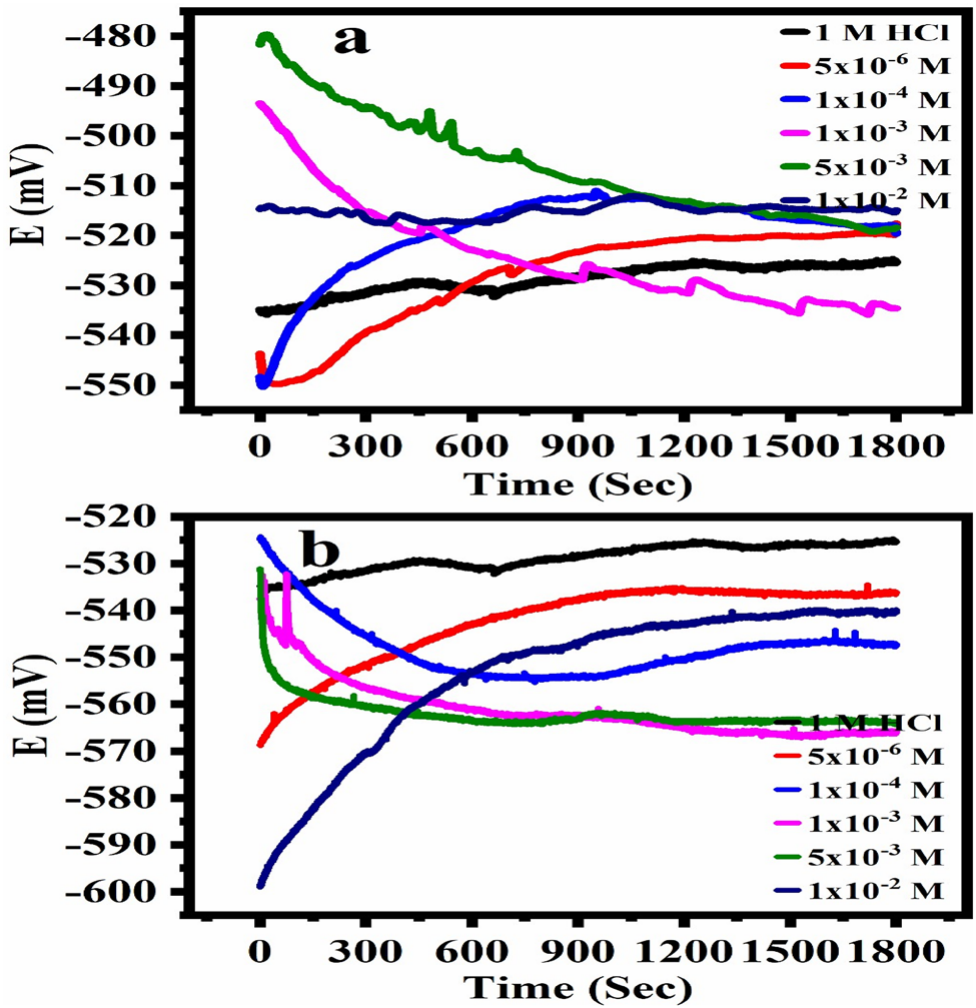
OCP–time curves for steel in 1.0 M HCl solution without and with different concentrations of DHSiMF (**a**) and DHSiB (**b**) at 30 °C.

Figure 3a and b shows the relationship between the potential and the time at a constant current in 1 M HCl in the absence and presence of the corrosion inhibitors DHSiMF and DHSiB, respectively. From Fig. 3a, we find that the behavior of DHSiMF concentrations differs at the time from 0 to 400 s. The potential of high concentrations start from less negative values then decreases until it reaches a steady state, while the low concentrations start from the more negative potential, then the potential increases until it reaches a steady state. It is also clear from Fig. 3a shows that the behavior of the inhibitor DHSiMF tends towards the cathodic direction concerning the blank, that is, the potential difference of the anticorrosion is greater than the voltage of the blank, and this indicates that the anticorrosion tends to prevent the cathodic reaction. On the other hand, it appears from Fig. 3b that the anticorrosion behavior of DHSiB at different concentrations is less in the anodic direction than the blank potential until it reaches a steady state, and this indicates that the anticorrosion prevents the anodic reaction. This behavior of corrosion inhibitors may be explained by the presence of furan and benzene rings with their contact with the methanol and hydroxyl groups, which work to increase and decrease the voltage in order.

### Electrochemical impedance measurements

**Figure 4:**
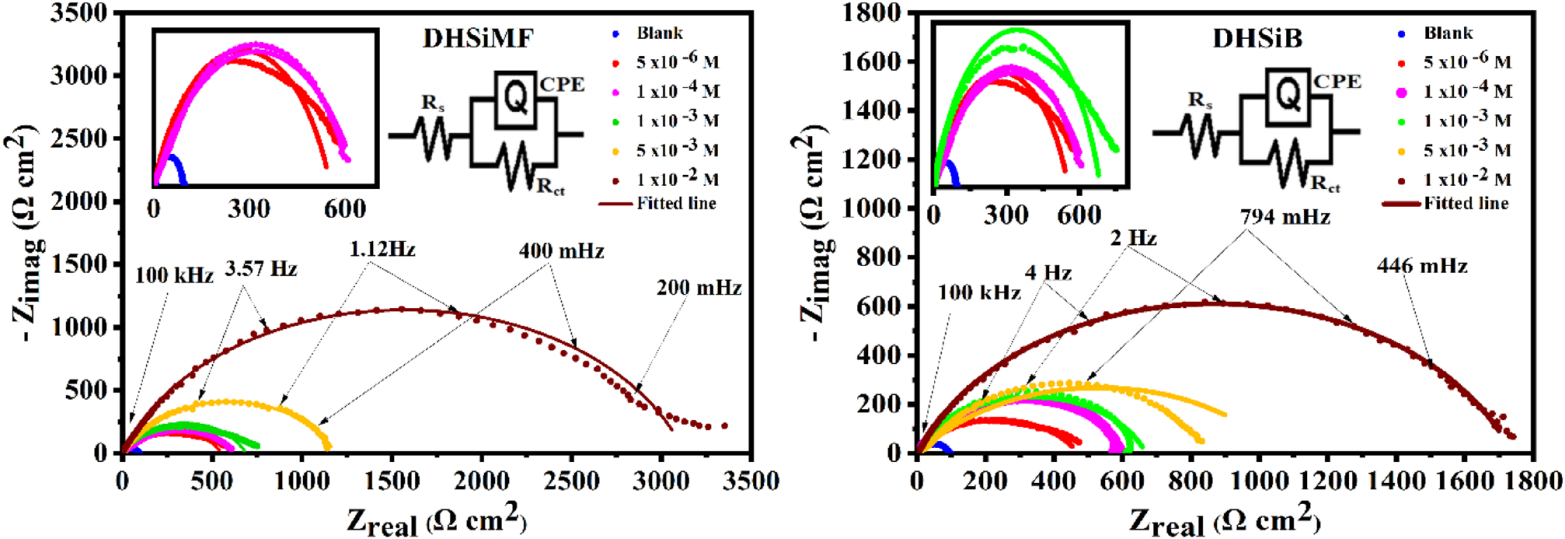
Nyquist plots for carbon steel in 1.0 M HCl in absence and presence of different concentrations of the (DHSiMF and DHSiB) compounds.

Parameters of electrochemical kinetic resulted by EIS-technique such as solution resistance (R_S_), charge transfer resistance (R_ct_), double layer capacitance (C_dl_), and inhibition efficiency (IE) evaluate the electrochemical properties of samples^35,36^. The impedance data of carbon-steel was clarified as Nyquist plots in Fig. 4 and reported in Table 2 after 30 min. as an inundation time in 1 M HCl with and without DHSiMF and DHSiB 10^–6^–10^–2^ mol L^–1^ concentrations at ambient temperature. At the higher and lower frequencies, the semicircles were produced, which cut the real axis. The solution resistance (Rs) is equal to the intercept produced from cutting the semicircles with the real axis at higher frequencies. While the summation of the solution resistance and charge transfer is equal to the intercept produced from cutting the semicircles with the real axis at lower frequencies. The difference between the two values at low and high frequencies equals charge transfer^37^. At the interface between the solution and metal, on the solution side, the ions govern the charge distribution, while on the metal side, the electrons govern the charge distribution. In other words, the charge of the metal will occupy a large volume on the solution side of the double layer because the ions are much larger than the electrons. So, CPE is used to talk about the non-ideal capacitive behavior of the double layer instead of C_dl_, which is the capacitance of the double layer ^38,39^. The capacitance of double layer (C_dl_) can be determined based on the following equation^40^:

6 $${C}_{dl}=Q{({\omega }_{max.})}^{n-1},$$

where $({\omega }_{max.}=2\pi {f}_{max.}$ and ${f}_{max.}$ are the frequencies at which the imaginary component of the impedance is maximal, respectively, and Q is a proportional factor (frequency independent constant). Figure 4 shows that the semi-circle diameter increases with the increase of the electrolyte concentration of DHSiMF and DHSiB detecting an enhancement in the resistance of corrosion.

**Table 2:** EIS parameters for the carbon steel in 1.0 M HCl in the absence and presence of different concentrations of the inhibitor compounds.

Inhibitor	Conc. (M)	R_s_ (Ω cm^2^)	R_ct_ (Ω cm^2^)	Q (μ Ω^–1^ s^n^ cm^–2^)	n	C_dl_ (μF cm^–2^)	Chi squared (χ^2^)	S	α°	θ	η_z_ %
Blank	–	4.699	91.21	1215.00	0.8632	857.368	3.34 × 10^–3^	− 0.572	− 55.20	–	–
DHSiMF	5 × 10^–6^	2.562	552	214.10	0.7165	91.971	9.89 × 10^–3^	− 0.640	− 56.79	0.835	83.48
	1 × 10^–4^	2.205	640.5	363.90	0.6297	154.538	1.76 × 10^–2^	− 0.570	− 51.29	0.858	85.76
	1 × 10^–3^	3.021	683.2	102.60	0.7777	47.995	8.87 × 10^–3^	− 0.701	− 63.75	0.866	86.65
	5 × 10^–3^	2.551	1183	105.40	0.7679	56.176	7.49 × 10^–3^	− 0.716	− 62.80	0.923	92.29
	1 × 10^–2^	2.816	3126	52.29	0.8024	33.474	6.95 × 10^–3^	− 0.770	− 66.78	0.971	97.08
DHSiB	5 × 10^–6^	2.626	468.4	341.70	0.674	140.852	1.13 × 10^–2^	− 0.573	− 52.08	0.805	80.53
	1 × 10^–4^	3.126	606.1	128.40	0.7774	61.808	6.26 × 10^–3^	− 0.676	− 60.57	0.850	84.95
	1 × 10^–3^	2.753	666	151.20	0.7697	76.076	8.38 × 10^–3^	− 0.671	62.10	0.863	86.30
	5 × 10^–3^	3.172	1059	469.60	0.5975	293.334	1.71 × 10^–2^	− 0.460	51.48	0.914	91.39
	1 × 10^–2^	2.953	1735	82.85	0.7818	48.214	5.82 × 10^–3^	− 0.724	− 65.67	0.947	94.74

Table 2 shows also the C_dl_ values at various DHSiMF and DHSiB concentrations. The electric capacitance of the electrode–electrolyte interface, or the rate of the charge/discharge process at the interface, is found to fluctuate in the presence of prepared inhibitors in the corrosive medium. As the concentration of the studied compounds rose, the C_dl_ values dropped. Furthermore, the capacitance outcomes may indicate an increase in the layer deposited by the DHSiMF and DHSiB concentration.

This is described by the creation of multiple layers of investigated inhibitors on the c-steel. The IE% values were estimated from Eq. (7)^41,42^:

7 $$IE\%=\left(1-\left[{R}_{ct}^{free}/{R}_{ct}^{inh.} \right]\right)\times 100,$$

where the charge transfer resistance in the free and inhibitor-containing states is symbolized as ${R}_{ct}^{free}$ and ${R}_{ct}^{inh.}$ respectively. It was found that the increase in DHSiMF and DHSiB rise the R_ct_ values determining a charge transfer resistance in the C-steel dissolution reaction^43^. The progressive removal of H_2_O reduced C_dl_ and the corrosion activity. The increase of IE values indicates the inhibiting influence of DHSiMF and DHSiB on the C-steel/solution interface is may due to the adsorption on surface with thin organo-silicon layers formation. The inhibition efficiency of DHSiMF of C-steel in 1 M HCl is more than DHSiB. Table 2 displays the n values derived from EIS measurements. The n value is around 0.86 when DHSiMF and DHSiB are absent. The formation of impurities, roughness, crystalline network flaws, active site size and irregular distribution, and other surface defects have all been connected to surface heterogeneity, which has been shown to be clearly visible at n values smaller than one^44^. The n values show that the presence of these chemicals impaired surface homogeneity because they were present in higher concentrations than those in the corrosive media without inhibitors. A more erratic metal-solution interface was produced by the adsorption of the DHSiMF and DHSiB, which have high molecular sizes and are asymmetric in relation to the corrosion products.

It is useful to get a hypothetical electrical equivalent circuit (EC) as mentioned above to investigate the frequency rate changes with electrochemical impedance^45^. As shown in Fig. 4, the EIS obtained from DHSiMF and DHSiB at different concentrations has been fitted to electrical EC. The literature has defined the EC of CPE parallel to (R_ct_), which was used to represent the iron/acid contact, and the analogous circuit for the suppression of steel acid corrosion^46^. Double layer capacitors are equiltative with constant phase elements (CPE) and some pore quantities are similar n quantities to 0 ˃ n < 1. Due to the charged surfaces being covered and the capacitive effects being diminished, the addition of (DHSiMF and DHSiB) causes the CPEs to drop.

**Figure 5:**
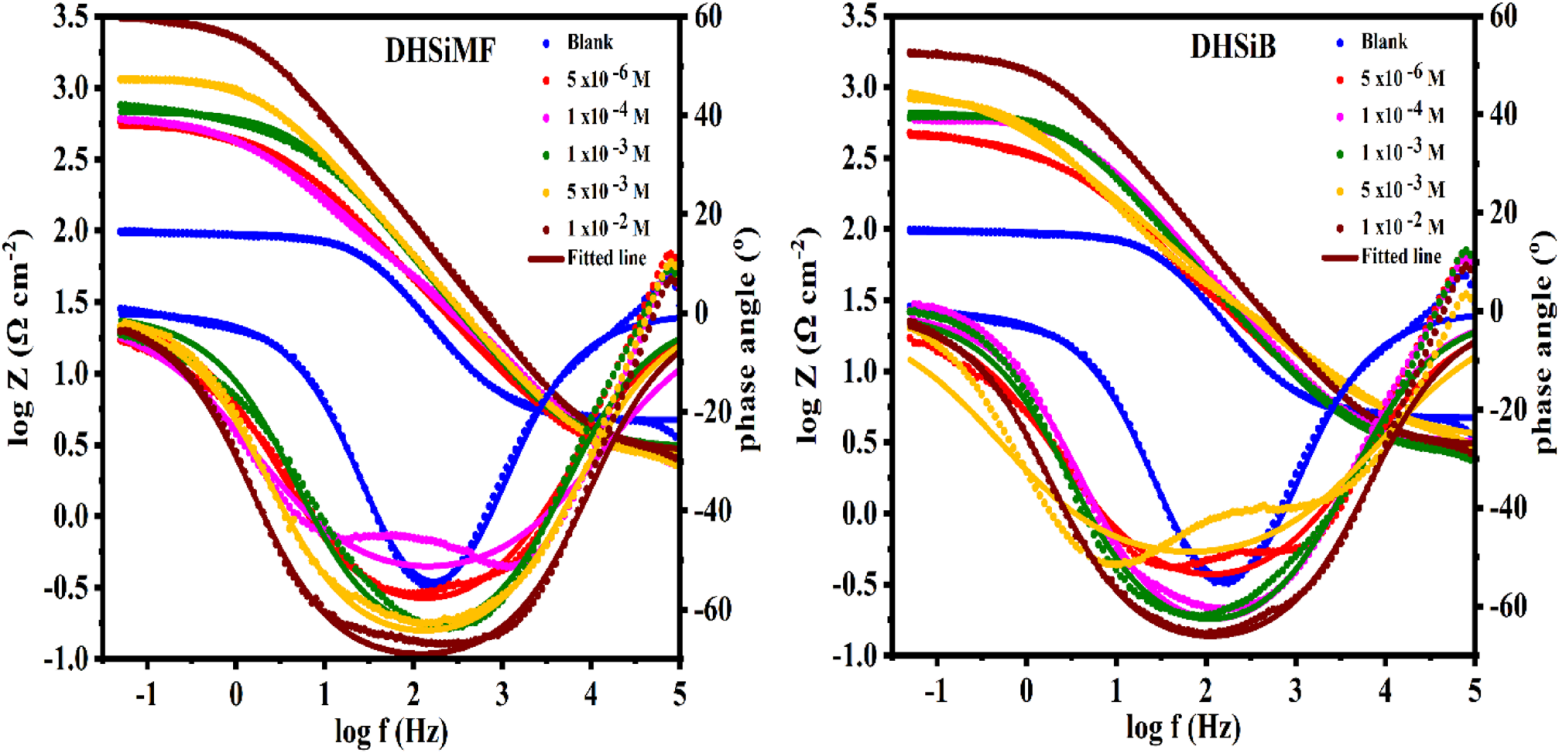
Bode and phase angle plots for carbon steel in 1.0 M HCl in absence and presence of different concentrations of the (DHSiMF and DHSiB) compounds.

For the Bodes plots (Fig. 5), the values of impedance at 0.01 Hz are important measurable parameter that may be used to evaluate the resistance to corrosion in the presence of inhibitors^47^. The strong corrosion resistance propensity of the PMS treated alloys is shown by high values of |Z|_0.01_ Hz. It is clear from Fig. 5 that the results obtained from |Z|_0.01_ Hz agree well with the Nyquist plots. Additionally, the presence of DHSiMF and DHSiB in the corrosive solution exhibit excellent corrosion resistance as evidenced by the high impedance level |Z| in the low frequency region^48^. For a pure capacitor, the slopes of lines at middle frequencies are − 1. In our situation, the improved capacitive behavior is indicated by the slopes shifting towards – 1 following the addition of DHSiMF and DHSiB molecules according to S values in Table 2. In contrast to the blank sample, the DHSiMF and DHSiB containing solutions have the greatest phase angle (α°) values, which achieved − 66.78 in case of highest concentration of DHSiMF. Since the phase angle value (α°) for perfect capacitive behavior is − 90°, this demonstrates the stability and insulation of the passivation and therefore the growth of capacitance behavior by addition of the inhibitor’s molecules^49^.

### Potentiodynamic polarization measurements

**Figure 6:**
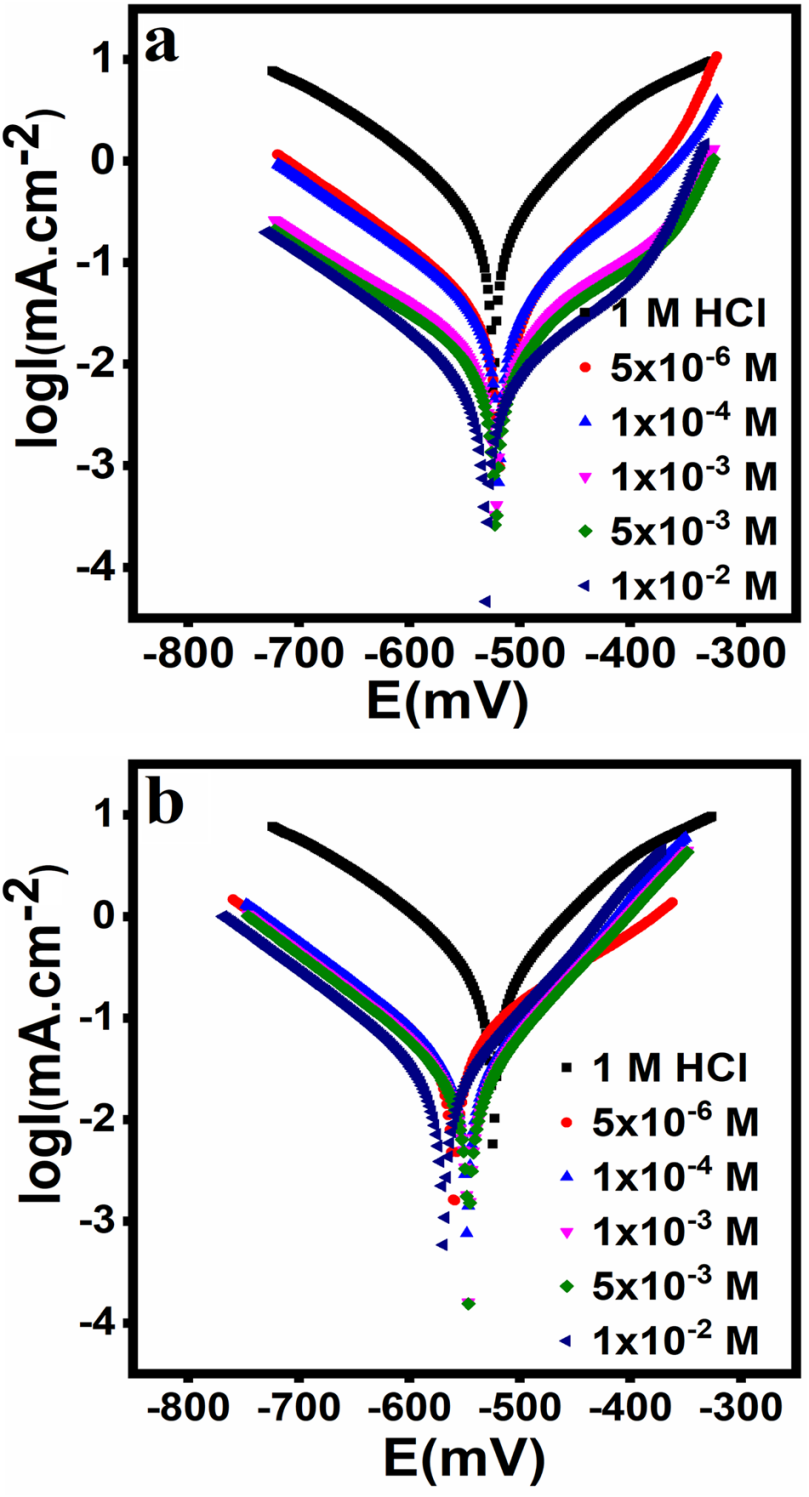
Potentiodynamic polarization curves for the corrosion of Steel in 1.0 M HCl in absence and presence of different concentrations of (DHSiMF and DHSiB).

Figure 6 shows the Potentiodynamic polarization curves for solutions of 1 M HCl with and without DHSiMF and DHSiB. The cathodic curves do not clearly demonstrate of Tafel regions therefore, the linear sections of the anodic regions have been used to determine the Potentiodynamic characteristics^50^. Tafel plots were used to evaluate the corrosion properties provided in Table 3: anodic and cathodic Tafel slopes (β_a_ and β_c_), inhibition efficiency (IE %), surface coverage degree (θ), corrosion current density (i_corr_), and corrosion potential (E_corr_). The polarization diagram shows the actions of the Tafel form.

**Table 3:** Polarization parameters for carbon steel in 1 M HCl in the absence and presence of different concentrations of the inhibitor compounds.

Inhibitor name	Conc (M)	E_corr_ vs.SCE (mV)	R_p_ (ohm.cm^2^)	i_corr_ (mA cm^–2^)	β_a_ (mV dec^–1^)	β_c_ (mV dec^–1^)	CR (mm/Y)	θ	η_PDP_ %
Blank	–	− 524	74	0.308	102	− 117	3.64	–	–
*DHSiMF*	5 × 10^–6^	− 518	617	0.029	86	− 108	0.347	0.9047	90.47
	1 × 10^–4^	− 519	994	0.026	90	− 110	0.308	0.9154	91.54
	1 × 10^–3^	− 515	1080	0.015	123	− 114	0.172	0.9527	95.27
	5 × 10^–3^	− 522	1592	0.008	95	− 113	0.094	0.9742	97.42
	1 × 10^–2^	− 513	3336	0.006	118	− 113	0.071	0.9805	98.05
*DHSiB*	5 × 10^–6^	− 559	470	0.045	135	− 132	0.527	0.8552	85.52
	1 × 10^–4^	− 547	695	0.034	88	− 127	0.406	0.8885	88.85
	1 × 10^–3^	− 569	680	0.028	132	− 144	0.336	0.9077	90.77
	5 × 10^–3^	− 546	908	0.025	88	− 124	0.297	0.9184	91.84
	1 × 10^–2^	− 564	1010	0.021	87	− 151	0.245	0.9327	93.27

Table 3 shows the (β_a_ and β_c_) values of that are not inhibitors concentration features and do not give a well-defined character. It also identifies the existence of DHSiMF and DHSiB in HCl medium not only decreases the anodized oxidation of C-steel but also slows down the cathodic hydrogen evolution. Both the cathodic and anodic overvoltage changed due to the presence of DHSiMF and DHSiB, this largely created a parallel displacement to the more negative and positive values connected with the blank curve^51,52^.

A further indication that DHSiMF and DHSiB molecules adsorb to the surface and lower the reaction after blocking the carbon steel surface reaction sites without altering the anodic and cathodic reaction mechanism is the small shift that the DHSiMF and DHSiB generate in (β_a_ and β_c_). From Eqs. (8), (9), the inhibition efficiency was computed^53,54^:

8 $${IE}_{icorr}\%=\left(1-\left[{i}_{corr.}^{inh,}/{i}_{corr.}^{free.} \right]\right)\times 100,$$

9 $${IE}_{CR}\%=\left(1-\left[{CR}_{corr.}^{inh,}/{CR}_{corr.}^{free.} \right]\right)\times 100,$$

where the corrosion current densities expressed by (i_corr_, and i_corr_) while the corrosion rate symbolized in (CR_corr,_ and CR_corr_) after and before inhibitor addition respectively. It is clear from Table 3 values that i_corr_ drops significantly for the studied media when DHSiMF and DHSiB are present, indicating that compounds adsorb on the surface thus suppressing both reduction of oxygen and dissolution of metals. It’s also reported that the E_corr_ values of the non-inhibitor systems have changed with the introduction of DHSiMF and DHSiB to the corrosive medium. Corrosion potential relocations (ΔE_corr_) caused by the existence of the tested inhibitors were determined according to the following equation:

10 $${\Delta E}_{corr}={E}_{corr}^{inh}-{E}_{corr}^{o},$$

where the corrosion potentials of the sample before and after the inhibitor, respectively, are ${E}_{corr}^{inh.}$ and ${E}_{corr}^{o.}$. Several papers have demonstrated that the E_corr_ value can also be used to determine the corrosion inhibition effect. If the E_corr_ values are less than 85 mV, the DHSiMF and DHSiB are considered inhibitors of mixed type. The DHSiMF and DHSiB can be either anodic or cathodic, depending on whether the E_corr_ is greater than + 85 mV or less than – 85 mV^55^. According to E_corr_ values, DHSiMF for the current work may be classified as a mixed-type inhibitor and DHSiB as an anodic inhibitor. While the amount of additives was increased, the corrosion rate (i_corr_) went down and the efficiency of stopping corrosion (% IE) went up. This shows that DHSiMF and DHSiB work as corrosion inhibitors on carbon steel in a 1 M HCl solution.

### Weight loss measurements

Table 4 lists the values of (CR_,_ mg cm^–2^ h^–1^), (Ө) and (% IE) for various concentrations of DHSiMF and DHSiB at 303, 313, 323, and 333 K. The data clearly show that as DHSiMF and DHSiB levels are increased and CR values are decreased, weight loss reduces, and at higher temperatures, the (%IE) increases. Once more, it appears that electrochemical measures may be relied upon to accurately predict the order of inhibitors’ efficacy in weight loss tests. The presence of benzene rings, Si, S, N, and O atoms in DHSiMF and DHSiB contributes to the %IE of carbon steel. Table 4 further shows that rising temperatures have an impact on CR and %IE values.

**Table 4:** Corrosion parameters obtained from weight loss measurements of carbon steel after 24 h immersions in 1 M HCl with and without addition of different concentrations of inhibitor compounds.

T (K)	Inhibitor conc. (M)	DHSiMF				DHSiB			
		ΔW (mg)	CR, (mg cm^-2^ h^–1^)	*θ*	*η*_*w*_* (%)*	ΔW (mg)	CR, (mg cm^–2^ h^–1^)	*θ*	*η*_*w*_*(%)*
303	Blank	93	0.4020	**–**	**–**	93	0.4020	**–**	**–**
	5 × 10^–6^	18	0.0778	0.806	80.65	22	0.0951	0.763	76.34
	1 × 10^–4^	16	0.0692	0.828	82.80	20	0.0865	0.785	78.48
	1 × 10^–3^	13	0.0562	0.860	86.02	18	0.0778	0.806	80.65
	5 × 10^–3^	12	0.0519	0.871	87.10	16	0.0692	0.828	82.79
	1 × 10^–2^	11	0.0475	0.882	88.17	14	0.0605	0.850	84.95
313	Blank	121	0.5230	**–**	**–**	121	0.5230	**–**	**–**
	5 × 10^–6^	34	0.1470	0.719	71.90	38	0.1643	0.686	68.59
	1 × 10^–4^	32	0.1383	0.736	73.55	35	0.1513	0.711	71.07
	1 × 10^–3^	28	0.1210	0.769	76.86	30	0.1297	0.752	75.20
	5 × 10^–3^	27	0.1167	0.777	77.69	28	0.1210	0.769	76.86
	1× 10^–2^	26	0.1124	0.785	78.51	25	0.1081	0.793	79.33
323	Blank	154	0.6657	**–**	**–**	154	0.6657	**–**	**–**
	5 × 10^–6^	57	0.2464	0.630	62.99	65	0.2810	0.578	57.79
	1 × 10^–4^	55	0.2377	0.643	64.29	60	0.2594	0.610	61.03
	1 × 10^–3^	51	0.2205	0.669	66.88	56	0.2421	0.636	63.63
	5 × 10^–3^	49	0.2118	0.682	68.18	52	0.2248	0.662	66.23
	1 × 10^–2^	48	0.2075	0.688	68.83	50	0.2161	0.675	67.54
333	Blank	162	0.7003	**–**	**–**	162	0.7003	**–**	**–**
	5 × 10^–6^	75	0.3242	0.537	53.70	80	0.3458	0.506	50.62
	1 × 10^–4^	73	0.3155	0.549	54.94	78	0.3372	0.518	51.85
	1 × 10^–3^	69	0.2983	0.574	57.41	74	0.3199	0.543	54.32
	5 × 10^–3^	68	0.2939	0.580	58.02	70	0.3026	0.568	56.79
	1 × 10^–2^	67	0.2896	0.586	58.64	68	0.2939	0.580	58.03

### Adsorption considerations

**Figure 7:**
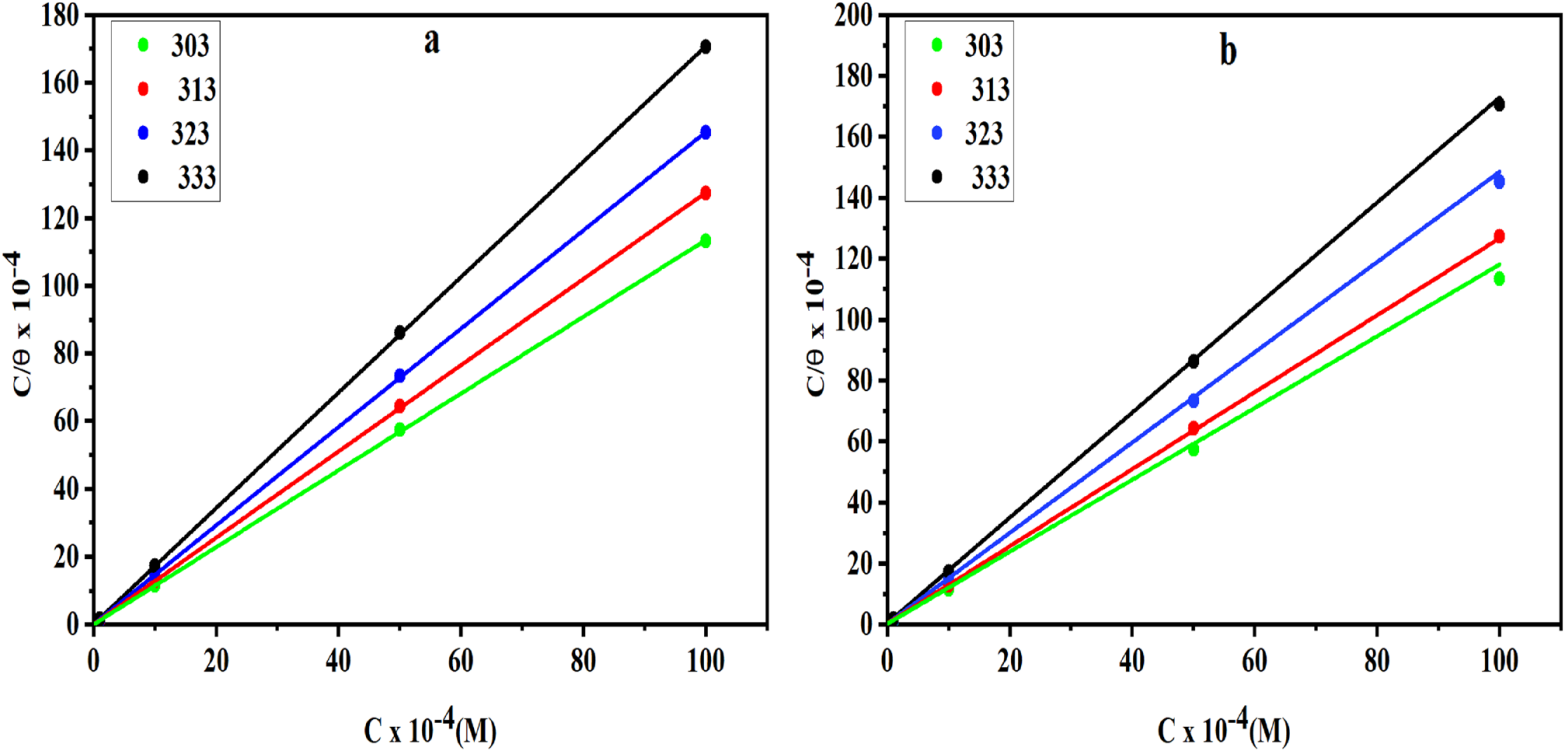
Langmuir isotherm [C/$\theta$] vs. [C] of ((**a**)-DHSiMF and (**b**)-DHSiB) inhibitors for carbon steel in 1 M HCl at different temperatures.

Adsorption isotherms can be used if the inhibitors’ efficacy is attributable to adsorption on the metallic substrate, as proven by electrochemical and weight loss data^56^. Adsorption isotherms are commonly acknowledged to explain the nature of the interaction between of active metal surface sites with inhibitor molecules^57^. To determine the covered surface (θ) as a function of inhibitor concentration (C_inh_), weight loss tests were utilized, which had already been graphically assessed by the several isotherms that are appropriate to pick the optimal convenient presented in this report. Based on R^2^ values closest to unity, the Langmuir isotherm has offered the finest overview of carbon steel adsorption and may be described as:

11 $${C}_{ihb}/\theta =1/{K}_{ads.}+{C}_{ihb},$$

where ${K}_{ads.}$ is the equilibrium adsorptive constant. The DHSiMF and DHSiB adsorption on the metal surface in HCl conforms to the Langmuir isotherm depending on the relationship of ${C}_{ihb}/\theta$ vs ${C}_{ihb}$ in Fig. 7.

The Langmuir isotherm argues that a fixed fraction of active locations on a solid surface contain a single adsorbed species. The equation depicts the relationship between ${K}_{ads.}$_._ and the standard free energy of adsorption ΔG_ads_ is^58^:

12 $${K}_{ads. }=\frac{1}{55.5}\mathrm{exp}\left(-\frac{\Delta {G}_{ads.}^{o}}{RT}\right),$$

where R is the universal gas constant and T is the absolute temperature. (55.5) is the concentration of water in solution mol L^–1^.

Table 5 illustrated the high ${K}_{ads.}$ and negative $\Delta {G}_{ads.}^{o}$ values which show that DHSiMF and DHSiB molecules are spontaneously and intensively adsorbed to the metal surface. The adsorption process will be compatible with the electrostatic interaction between charged molecules and the charged metal surface (physic-sorption) if thermodynamic calculations yield $\Delta {G}_{ads.}^{o}$ values of – 20 kJ mol^–1^. Charge transfer from DHSiMF and DHSiB molecules to the metal surface occurs during chemisorption^59^ at $\Delta {G}_{ads.}^{o}-$40 kJ mol^–1^. For this study, the values of $\Delta {G}_{ads.}^{o}$ are between-37.51 kJ mol^–1^ and − 34.42 kJ mol^–1^ for DHSiMF and DHSiB at temperatures ranging from 303 to 333 K. The physical and chemical adsorption are thus thought to be the best explanation for the adsorption process for the complex interactions of DHSiMF and DHSiB on the carbon steel surface^60^. Table 5 further implies that chemical adsorption is more likely for DHSiMF and DHSiB in examined solutions, which may be confirmed by comparing the (%IE) for each inhibitor at the above-mentioned temperatures. The Van’t Hoff Eq. (13) enabled us to calculate the adsorption heat ($\Delta {H}_{ads.}^{o}$)^61,62^:

**Table 5:** Thermodynamic parameters for the adsorption of inhibitor compounds in 1 M HCl on carbon steel surface.

Inhibitor	T(K)	Slope	Intercept	R^2^	K_ads_ M^–1^	ΔG^o^_ads_ kJ/mol	ΔS^o^_ads_ J/mol.K	ΔH^o^_ads_ kJ/mol
DHSiMF	303	1.13430	0.00002	0.9999	49,881	− 37.37	12.55	− 7.90
	313	1.27428	0.00002	0.9999	52,769	− 37.51	12.15	
	323	1.45333	0.00002	0.9999	42,148	− 36.94	10.91	
	333	1.70702	0.00003	0.9999	39,165	− 36.76	9.23	
DHSiB	303	1.17786	0.00004	0.99983	23,971	− 35.52	84.76	− 11.07
	313	1.26304	0.00005	0.99977	20,102	− 35.08	82.66	
	323	1.48153	0.00005	0.99990	19,800	− 35.04	81.65	
	333	1.72407	0.00006	0.99988	15,495	− 34.42	82.74	

13 $$ln{K}_{ads.}=-\frac{\Delta {H}_{ads}^{o}}{RT} +constant.$$

**Figure 8:**
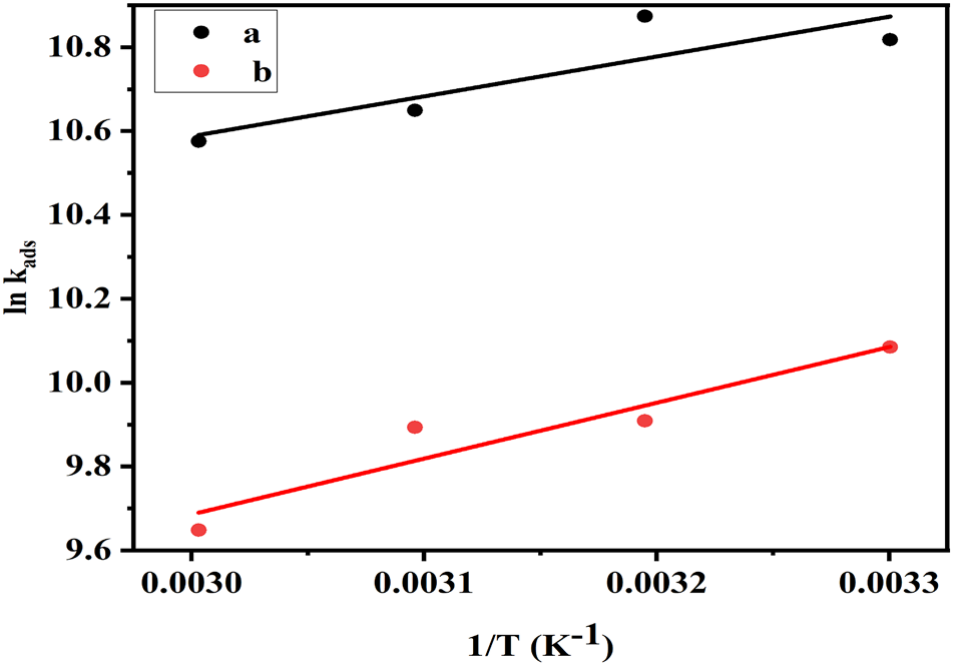
Van’t Hoff equation represents the relation between ln K_ads_ and 1/T for (DHSiMF (a) and DHSiB (b)) in 1.0 M HCl solution.

When $ln{K}_{ads.}$ and 1/T are plotted, $\Delta {H}_{ads}^{o}$ can be produced (Fig. 8). (− $\Delta {H}_{ads}^{o}$ /R) denotes the slope of the straight lines created.

Adsorption heat under test circumstances can be generally characterized as the standard heat of adsorption $\Delta {H}_{ads}^{o}$. Finally, ($\Delta {S}_{ads}^{o}$) the standard adsorption entropy may be calculated^62^ using the Eq. (12):

14 $$\Delta {G}_{ads.}^{o}=\Delta {H}_{ads.}^{o} -T\Delta {S}_{ads}^{o}.$$

The value of $\Delta {H}_{ads.}^{o}$ reveals more about the mechanism of corrosion inhibition. When ($\Delta {H}_{ads.}^{o}$ < 0), the adsorption process is followed by the release of heat (an exothermic process), which can be chemi-sorption, physic-sorption, or both, whereas the endothermic process is associated with chemi-sorption^63^. The values of $\Delta {H}_{ads.}^{o}$ may also be used to forecast whether the exothermic process is chemi-sorption or physic-sorption. Adsorption can be divided into two types: physic-sorption ($\Delta {H}_{ads.}^{o}$− 40 kJ mol^–1^) and chemi-sorption ($\Delta {H}_{ads.}^{o}$− 100 kJ mol^–1^)^64^. Table 5 displays the thermodynamic values obtained. The values of $\Delta {H}_{ads.}^{o}$ indicate that inhibitor adsorption is an exothermic process, with IE% decreasing as temperature rises.

### Thermodynamic activation parameters

The findings in Table 6 show that (CR) and (IE %) are directly and inversely proportional to temperature respectively, with absence and presence of DHSiMF and DHSiB. This is possibly because of the reduced rate of adsorption of DHSiMF and DHSiB compounds on the metal surface at elevated temperatures. The Arrhenius Eq. (15) represents the relation between the corrosion rate of the metal (CR) and temperature (T)^65^.

15 $$CR=A\,exp \left(-\frac{{E}_{a}}{RT}\right),$$

where Ea, A, and R are the apparent activation energy, frequency factor, and gas constant (8.314) J K^–1^ mol^–1^.

**Table 6:** Activation parameters of dissolution reaction of carbon steel in 1 M HCl with at different concentrations of inhibitor compounds.

Inhibitor	Conc. of inhibitors (M)	E_a_ (kJ mol^–1^)	∆H* (kJ mol^–1^)	∆S* (J mol^–1^ K^–1^)
Blank	0.00 × 10^–4^	16.09	13.17	– 207.90
DHSiMF	5 × 10^–6^	40.42	36.99	− 141.04
	1 × 10^–4^	42.91	39.43	− 133.68
	1 × 10^–3^	47.91	43.68	− 120.99
	5 × 10^–3^	48.86	45.26	− 116.29
	1 × 10^–2^	50.89	47.24	− 110.25
DHSiB	5 × 10^–6^	37.15	33.78	− 150.29
	1 × 10^–4^	38.89	35.50	− 145.37
	1 × 10^–3^	40.91	37.47	− 139.80
	5 × 10^–3^	42.44	38.97	− 135.66
	1 × 10^–2^	45.70	42.16	− 126.04

**Figure 9:**
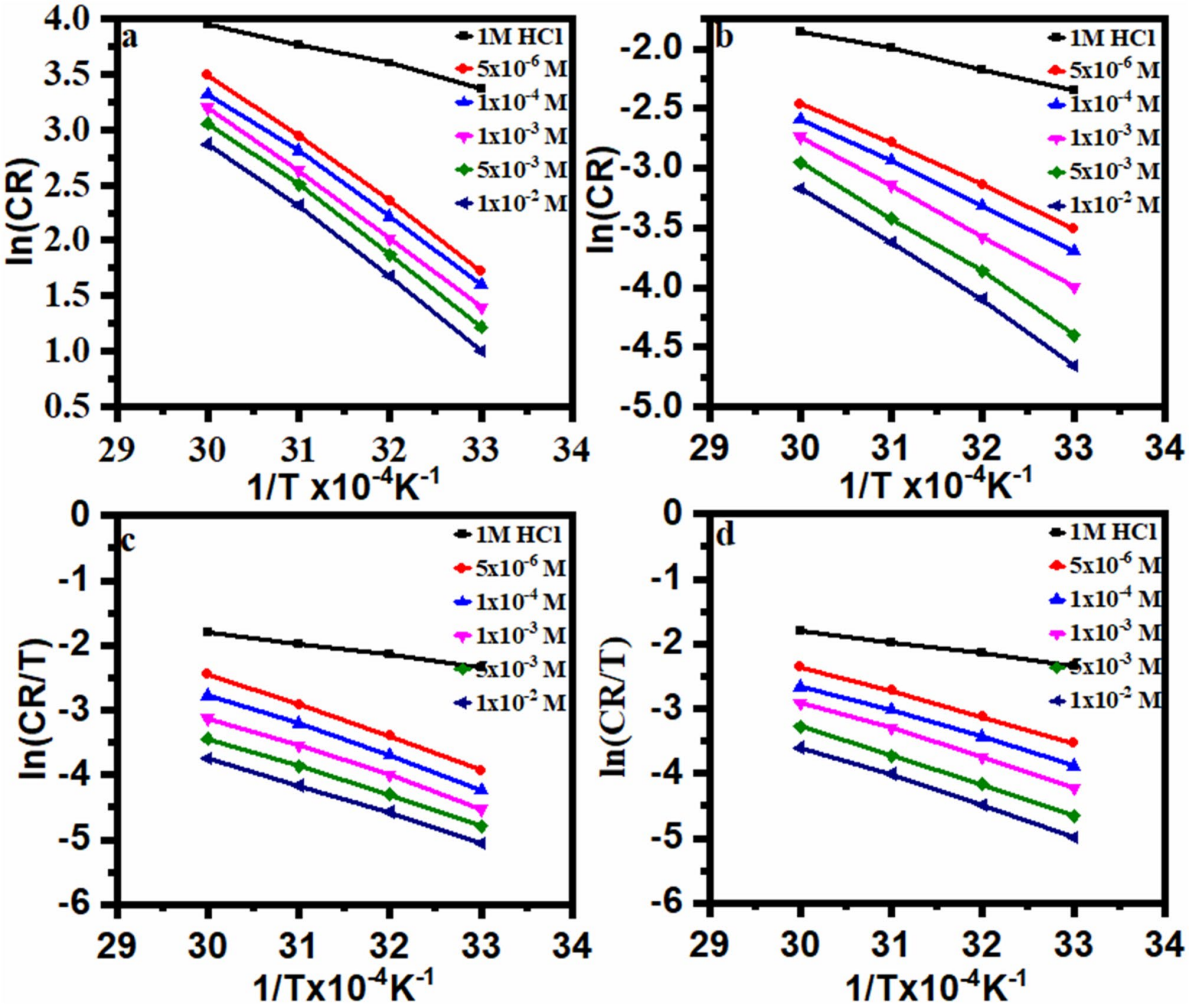
Arrhenius plots and Transition state plots for carbon-steel dissolution with and without different concentrations of (DHSiMF and DHSiB) in 1.0 M HCl solution.

The plot between an ln CR vs 1/T for carbon steel in the tested HCl medium is shown in Fig. 9, both after and before the addition of varied inhibitor doses. Table 6 shows the data for calculating E_a_ using the derived regression equation between ln CR and 1/T. Each straight-line slope denotes a different value of (E_a_). Table 6 shows that the Ea values achieved in the absence and presence of an inhibitor (DHSiMF and DHSiB) were 16.09, 50.89, and 54.70 kJ mol^–1^, respectively. The greater energy barrier for the process of dissolving steel and the adsorbed DHSiMF and DHSiB layer on the surface of the steel, which slows down the process of charge/mass transfer, are the two causes of the rise in Ea values in the presence of DHSiMF and DHSiB^66^. Due to the formation of a steel-Schiff bases complex or a slowdown in the rate of steel dissolution, Ea values grew as organic inhibitors concentration rose^67^. The transition state equation was used along with an extra version of the Arrhenius equation^68^:

16 $$CR=\frac{RT}{Nh}\mathrm{exp}\left(\frac{\Delta {S}_{a}^{o}}{R}\right)-\mathrm{exp}\left(-\frac{\Delta {H}_{a}^{o}}{RT}\right),$$

where h is the Plank constant (6.626176 × 10^–34^Js) and N denotes Avogadro’s number (6.02252 × 10^23^ mol^–1^). The plot draw of ln CR/T vs 1/T for metal corrosion before and after the addition of DHSiMF and DHSiB is shown in Fig. 9. Table 6 lists the enthalpy of activation ($\Delta {H}_{a}^{*}$) and entropy of activation ($\Delta {S}_{a}^{*}$) which were calculated from the slope and intercept of straight lines, respectively. It was found that ($\Delta {H}_{a}^{*}$) values for DHSiMF are positive, which refers to the endothermic nature of the metal oxidation process, making it difficult to dissolve depending in the analyzing the results are given in Table 6. These results demonstrate that both $\Delta {H}_{a}^{*}$ and $\Delta {S}_{a}^{*}$ values in the presence of DHSiMF and DHSiB rise above those in the blank, indicating an increase in the energy barrier for corrosion reaction in the presence of DHSiMF and DHSiB^69^. The difficult and slow endothermic nature of the dissolving process was demonstrated by the positive values of $\Delta {H}_{a}^{*}$^70^. The molecules are orientated on the surface and ordered by adsorption through the active center, which results in a rise in the value of $\Delta {S}_{a}^{*}$ (less negative), which indicates a decrease in disorder^71^.

### Molecular dynamics and Monte Carlo simulations

**Figure 10:**
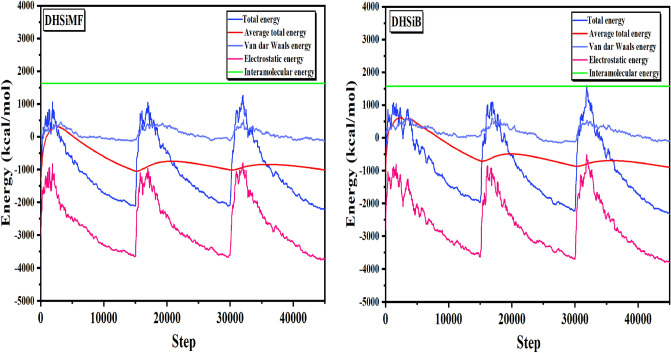
Distribution of the different energy terms during the process of optimization of the adsorption of for (DHSiMF and DHSiB (protonated)/200 H_2_O/19 H3O^+^/ 20 Cl^–^) systems onto the Fe (110) surface obtained by via MC.

**Figure 11:**
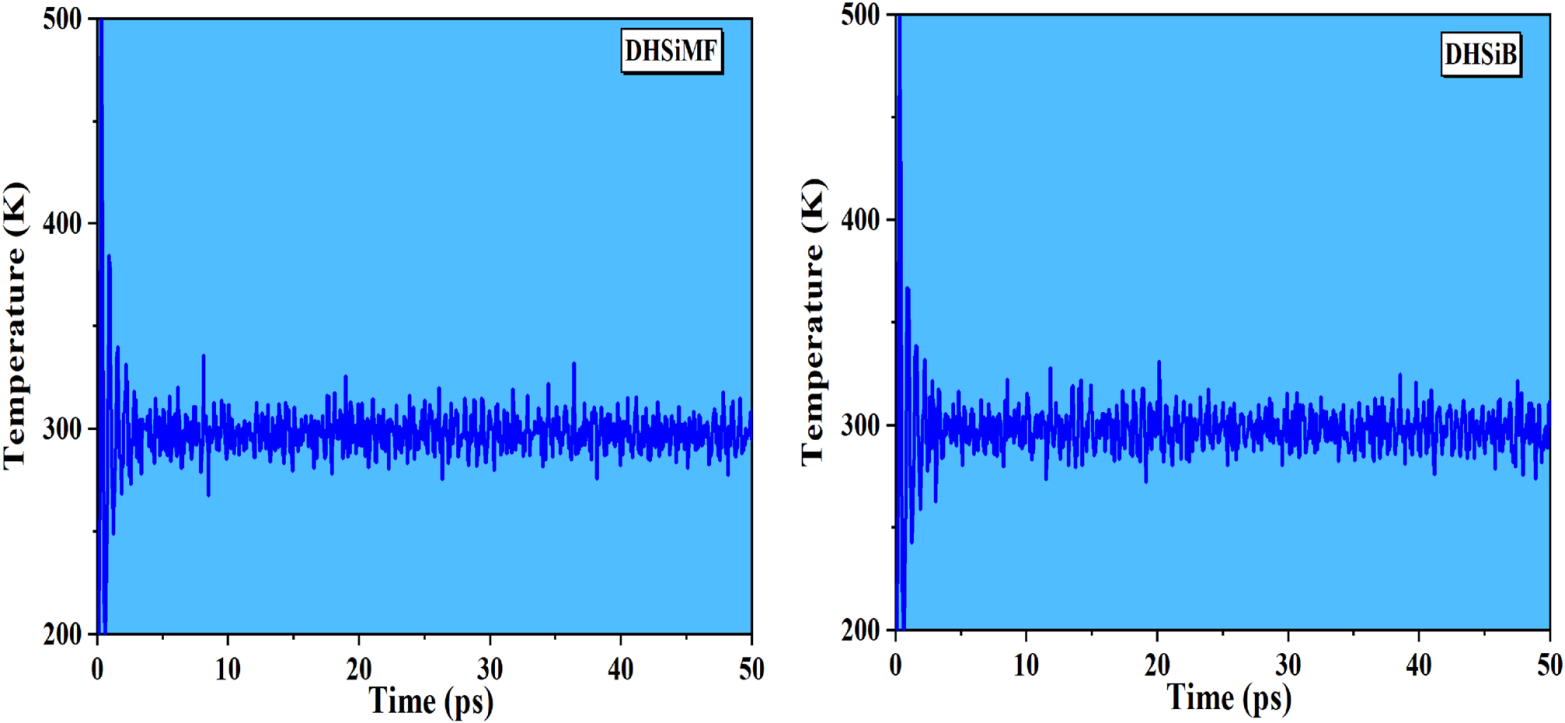
Temperature fluctuation at T = 298 K (DHSiMF and DHSiB (protonated)/200 H_2_O/19 H3O^+^/20 Cl^–^) systems onto the Fe (110) surface, obtained via MD.

In order to comprehend the inhibitory phenomena on the steel surface and identify the low E_ads_ locations on the surface, MC and MD simulations were carried out. This allowed identification of the preferred adsorption sites and geometry of DHSiMF and DHSiB compounds either in the neutral or protonated form^72–74^. By optimizing the entire system, different forms of energies for DHSiMF and DHSiB inhibitors on the Fe (1 1 0) surface in the simulated corrosive fluid were determined. The findings are shown in Fig. 10. Also, through the analysis of T (K) variations in MD simulation, the most stable adsorption sites of DHSiMF and DHSiB were found shown in Fig. 11. Figure 11 illustrates how little T(K) fluctuation there is, demonstrating the accuracy of our system’s MD estimates^75^.

**Figure 12:**
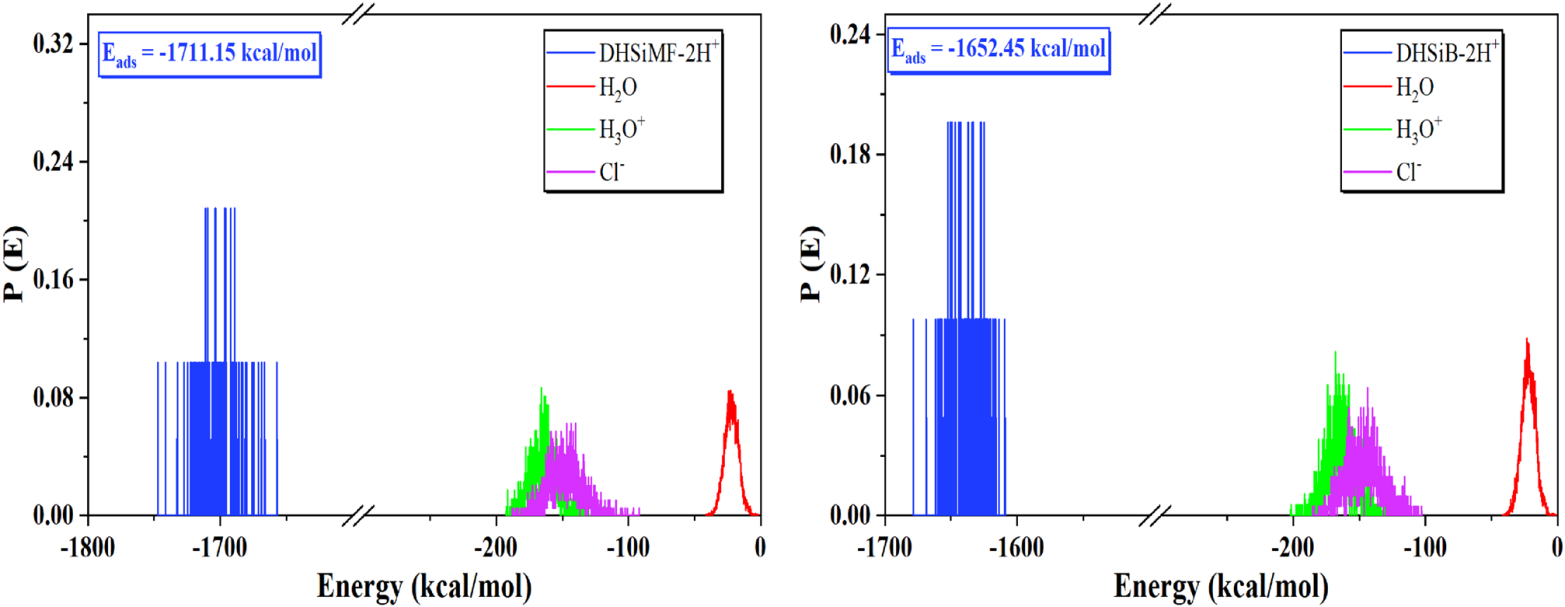
Distribution of the *E*_ads_ of the (DHSiMF and DHSiB (protonated)/200 H_2_O/19H_3_O^+^/20 Cl^–^) system via MC simulation.

The abundance of the E_ads_ for the examined compounds DHSiMF and DHSiB on the surface of Fe (110) substate is shown in Fig. 12. The values of the several types of adsorption energies (total, rigid, deformation) are collected in Table 7. The adsorption process is spontaneous, as indicated by the negative E_ads_ values. Compared to other molecules precent in then corrosive media, the DHSiMF and DHSiB molecules have substantially larger E_ads_ distributions. The E_ads_ distribution in Fig. 10 shows that the DHSiMF and DHSiB molecules may progressively substitute the adsorbed corrosive ions (H_3_O^+^, Cl^-^) and water molecules from the steel surface^76^. In simulated corrosion tests, the inhibitor E_ads_ on the steel sample follow this pattern: DHSiMF (− 1711.15 kcal/mol) > DHSiB (− 1652.45 kcal/mol).

**Table 7:** The outputs and descriptors calculated by the Monte Carlo simulations for adsorption of DHSiMF and DHSiB on Fe (110) (in kcal/ mol).

Phase	Inhibitor	Total energy (kcalmol^–1^)	Adsorption energy (kcalmol^–1^)	Rigid adsorption energy (kcalmol^–1^)	Deformation energy (kcalmol^–1^)	(dE_ads/dNi_) (kcalmol^–1^)	Binding energy (kcalmol^–1^)	IE* (%)
Gas phase	*DHSiMF*	− 320.190	− 2546.808	− 264.788	− 2282.020	− 2546.808	2546.808	98.05
	*DHSiB*	− 259.264	− 1651.987	− 218.506	1433.481	− 1651.987	1651.987	93.27
	*DHSiMF* –2H^+^	− 305.284	− 1720.765	− 251.223	− 1469.541	− 1720.765	1720.765	98.05
	*DHSiB* -2H^+^	− 241.076	− 1708.202	− 208.524	− 1499.677	− 1708.202	1708.202	93.27
Aqueous phase	*DHSiMF*	− 6018.713	− 7559.676	− 6090.718	− 1468.958	− 1568.675	1568.675	98.05
	*DHSiB*	− 6063.318	− 7615.510	− 6149.432	− 1466.078	− 1510.379	1510.379	93.27
	*DHSiMF* –2H^+^	− 5663.332	− 7292.963	− 5765.960	− 1527.003	− 1711.156	1711.156	98.05
	*DHSiB* -2H^+^	− 5747.798	− 7325.785	− 5819.061	− 1506.724	− 1652.457	1652.457	93.27

**Figure 13:**
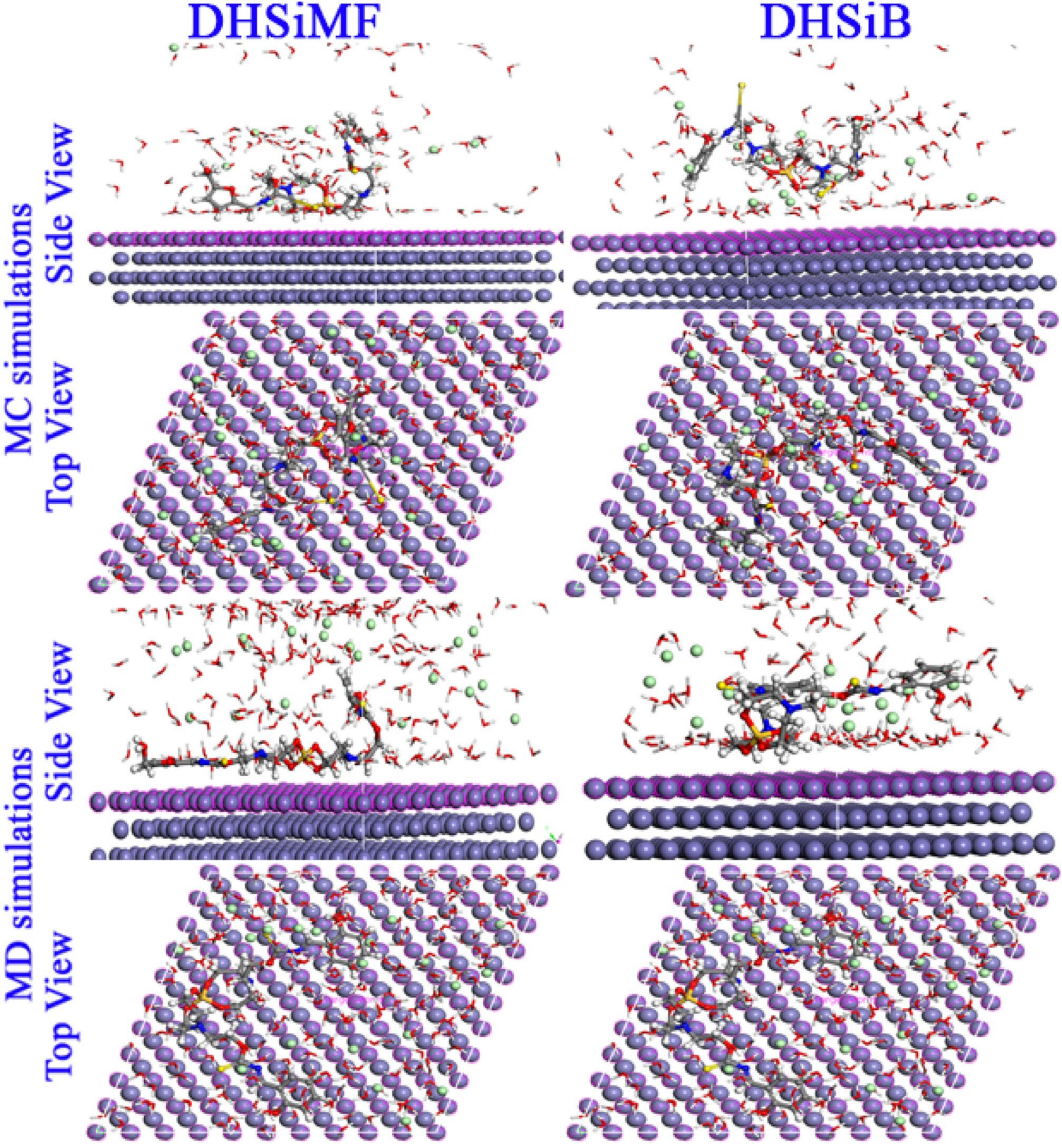
MC and MD simulations results for the most favorable modes of adsorption configurations and positions obtained for (DHSiMF -2H^+^ and DHSiB -2H^+^/200 H_2_O/19 H_3_O^+^/20 Cl^–^) on Fe (1 1 0) surface, side, and top view.

According to data in Table 7, DHSiMF and DHSiB molecules either in neutral or protonated forms can be ranked in order of their potency as inhibitors for the gas or aqueous phase adsorption: DHSiMF > DHSiB. The exceptionally high levels of binding energies show the DHSiMF and DHSiB molecules’ extraordinary ability to resist corrosion for steel^77,78^. The equilibrium configurations of both inhibitors’ adsorption on the surface of Fe (1 1 0) are depicted in Fig. 13. The fact that each inhibitor molecule is adsorbed on the surface of Fe (1 1 0) in almost parallel mode (position) of the inhibitor’s rigid structure with regard to the surface of the metal is clearly shown in Fig. 13, which supports the strong connection and extra surface coverage between the inhibitor and the Fe atoms^79,80^. By analysis of the molecular structures of DHSiMF and DHSiB, we can note that sharing the electrons of the N, O, S, and aromatic rings present in the two inhibitors can facilitate the adsorption on the surface of steel. This is done by forming a strong Fe–N, Fe–S, and Fe–O chemical bond between the molecules and the Fe (1 1 0) surface^81,82^ Also, the Van der Waals dispersion forces and electrostatic attraction can govern the physical interactions between the investigated inhibitors and the mild steel surface^83^.

**Figure 14:**
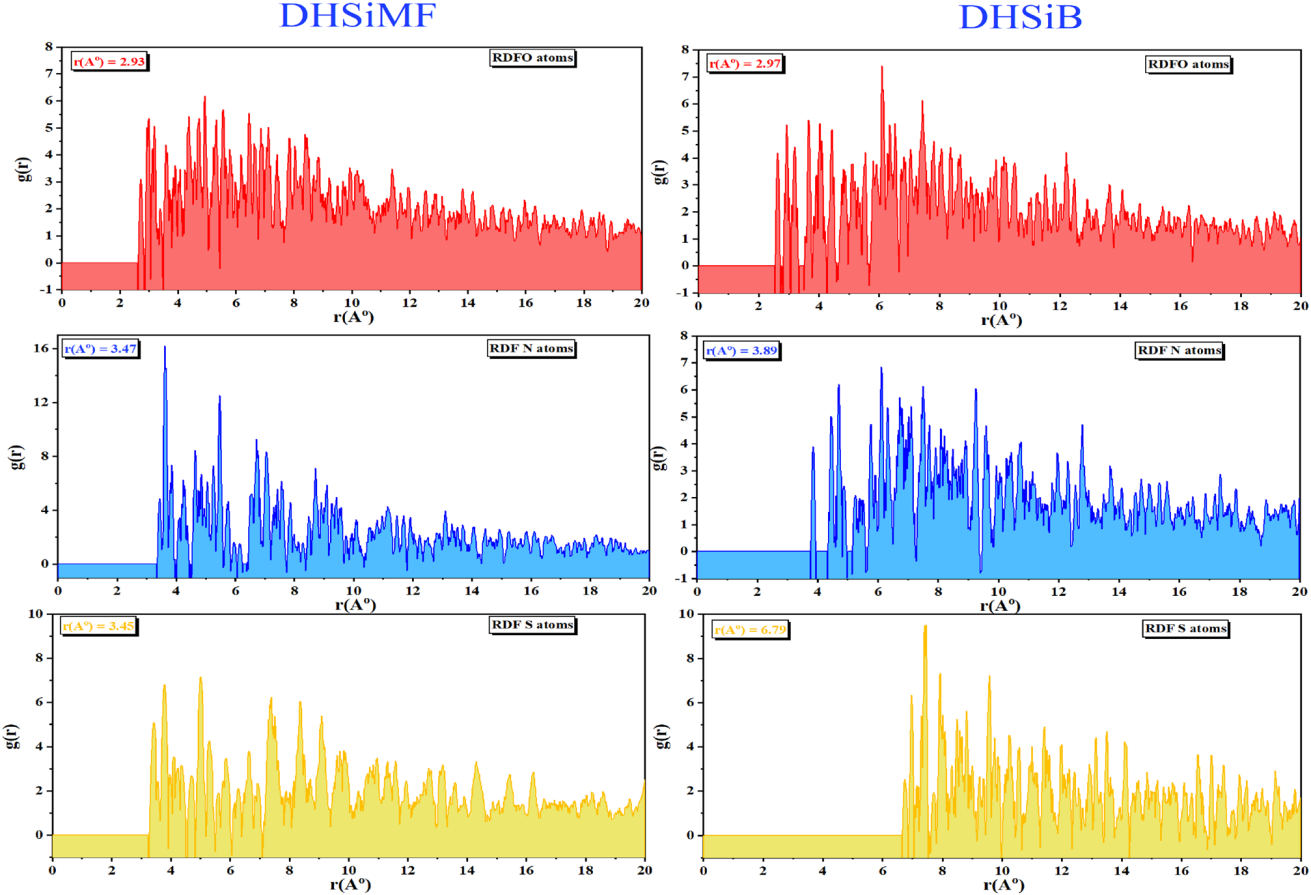
RDF of the O, N and S heteroatoms for DHSiMF and DHSiB inhibitors/Fe (110), obtained via MD.

The bond length between the Fe (110) and the atoms of the DHSiMF and DHSiB molecules was calculated using the radial distribution function (RDF) analysis of the MD data (Fig. 13). By determining the values of the bond lengths, the various types of bonds formed can be determined. The sort of adsorption activity taking place on the metal is described by peaks of the RDF graph that appear at specific distances from the metal surface^84,85^. The chemisorption mechanism is represented as a process when the peak is present between 1 and 3.5 Å, however for physisorption, the RDF peaks are expected to be present at distances larger than 3.5 Å^86,87^. DHSiMF and DHSiB molecules have relatively strong contact with the steel surface because of the close position of their heteroatoms to the steel surface, as seen in Fig. 14, which supports their reflected inhibitory performance^88^.

### SEM results

**Figure 15:**
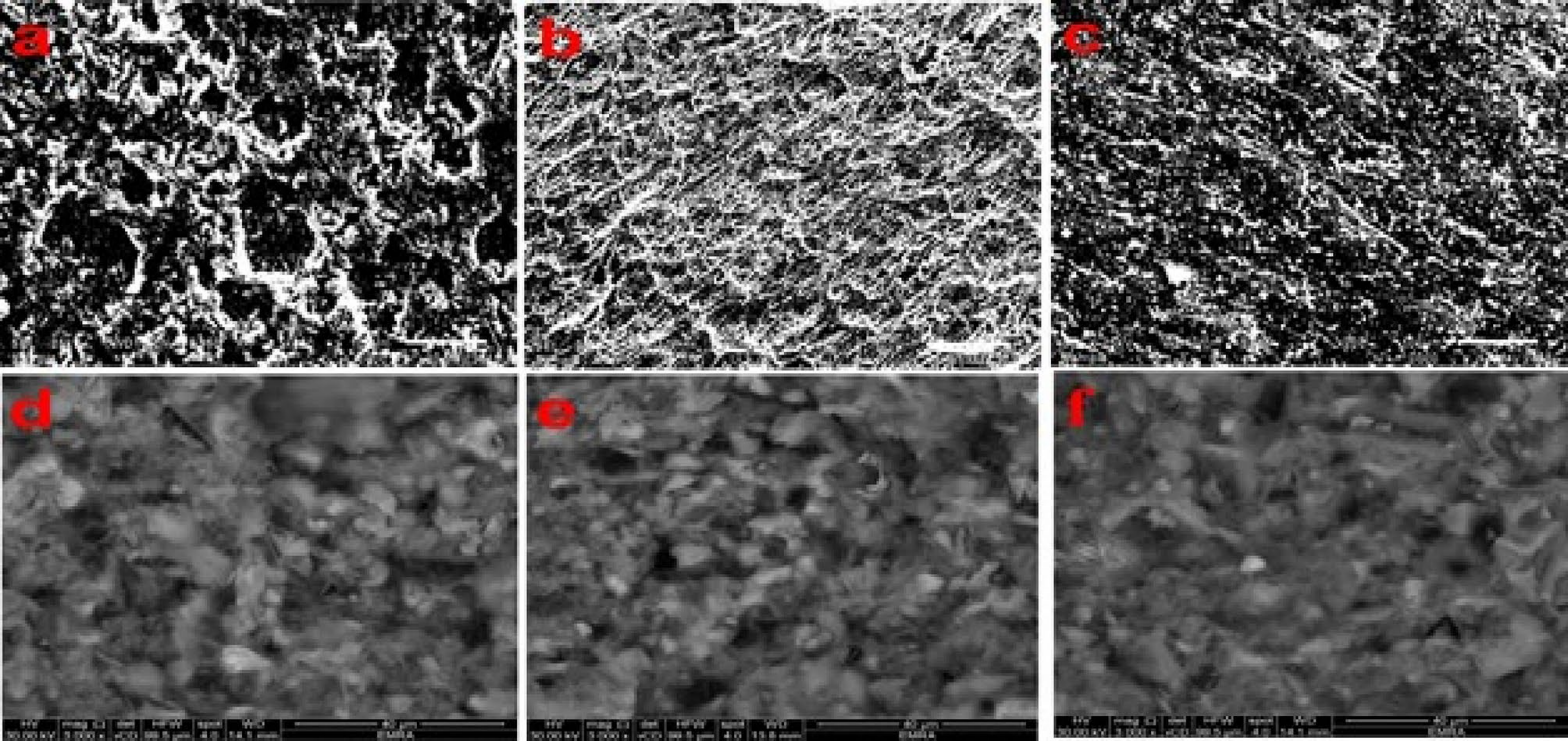
SEM images of carbon-steel and concrete specimen with and without (DHSiMF and DHSiB).

Figure 15 displays a variety of SEM pattern reported for C-steel specimen processed for 24 h in various media studied. The characterization of the C-steel surface immersed in tested HCl solution was seen in Fig. 15a. The images show that the metal is corroded with localized zones in the absence of investigated Schiff base. With addition of DHSiMF and DHSiB, (Fig. 15b,c), the rate of corrosion is suppressed causing a reduction in the region affected by corrosion and the protective layer covers much of the C-steel surface. The images show that when DHSiMF and DHSiB are present, a protective coating formed on the surface of carbon steel that makes it look like it almost doesn’t rust. It is also evident from the figure with the sample treated with x, the formation of more than one layer of inhibitor. This is logically consistent with the results of the chemical and electrochemical tests.

On the other hand, this study performed SEM analysis on samples after 28 days of curing to better understand the changes in internal micros morphology of concrete following the addition of (DHSiMF and DHSiB chemicals). Before the test, the sample is gold-plated to enhance its electrical conductivity, providing for a clearer image of the micromorphology. Several SEM images were produced in this investigation, and the best of them were chosen, as shown in Fig. 15d–f for concrete, without and with 400 ppm of DHSiMF, and DHSiB, respectively. Figure 15d shows that the concrete mixture free of amide compounds, consisting of fine and coarse aggregates, sand, cement, and water, was bonded by the formation of hydration bonds. These bonds covered the concrete aggregates in the form of ginned cotton. One can notice found the deep gaps between the coarse, fine aggregates, and sand. On the other hand, Figs. 15e,f for concrete blended with amide compounds is based on silaspiro group (DHSiMF and DHSiB) respectively. It has been found that from these figures, the hydration formation is increased due to the abundance of bond formation between cement and amide compounds. Furthermore, increasing the bond covered for the concrete aggregates in the form of ginned cotton and decrease the gaps between the aggregates.

### XRD results

The technique of XRD was employed to offer methodological validation of the inhibitory characteristics of synthesized compounds DHSiMF and DHSiB for carbon steel in 1 M HCl.

**Figure 16:**
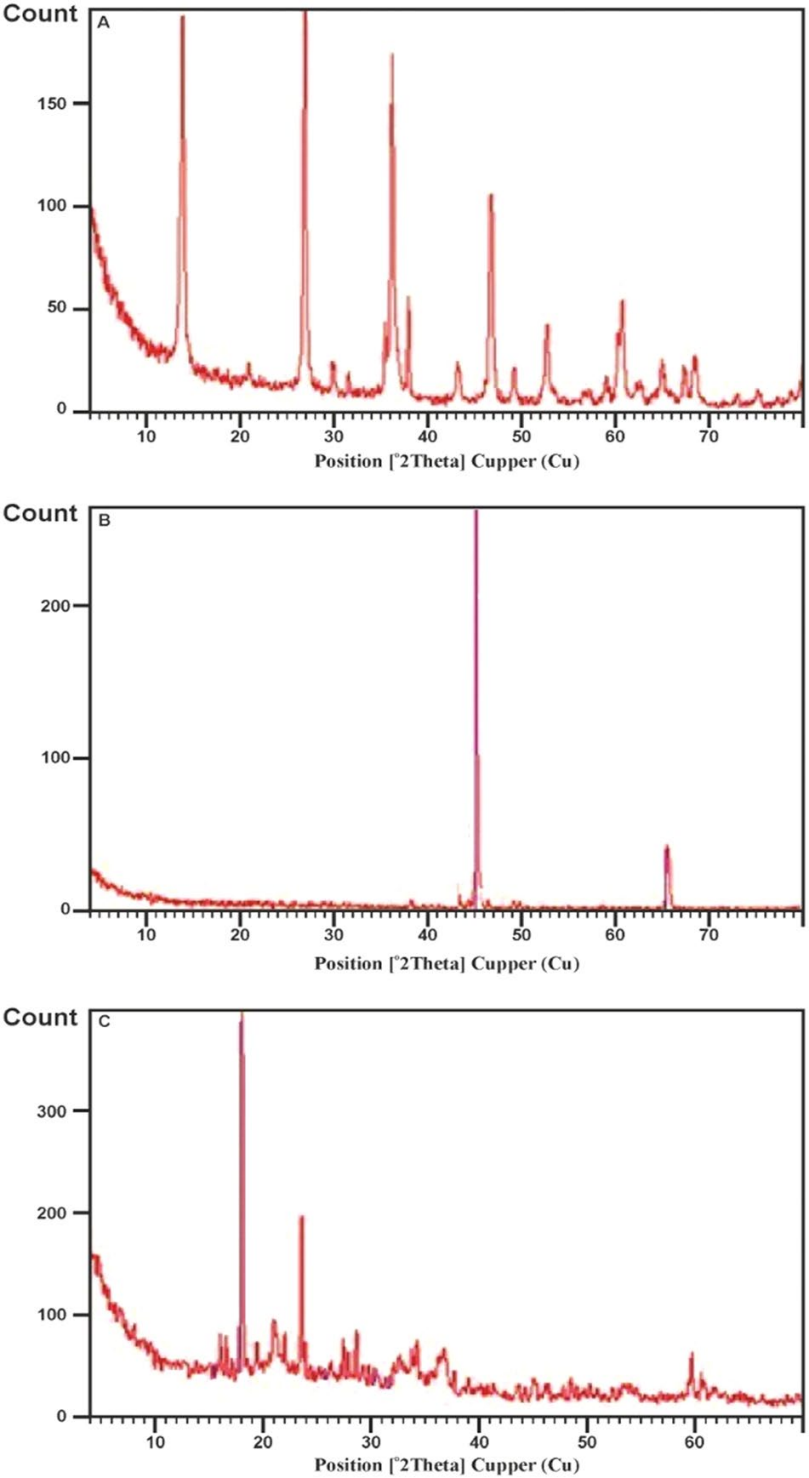
XRD images of carbon-steel specimen (1.0 M HCl, DHSiMF and DHSiB respectively) after 24 h immersion period.

The diagrams of XRD are shown in Fig. 16 for carbon steel immersion in 1 M HCl solution for 24 h without and with inhibitor compounds (DHSiMF and DHSiB). The production of magnetite peaks attributable to iron oxides (Fe3O4 and FeOOH) appearing at 2θ = 14.73°, 26.97°, 37.12°, 53.54°, and 61.34° due to the carbon steel surface obviously subjected to corrosion in the absence of an inhibitor. On the other hand, the iron maximum peak occurs at 2θ = 45.6°. XRD analysis revealed no magnetite on any of the specimens treated with DHSiMF and DHSiB. An oxide film on carbon steel will eventually form magnetite, which will convert from (γ -FeO(OH)) to goethite (α -FeO(OH)).

### Compressive strength

**Figure 17:**
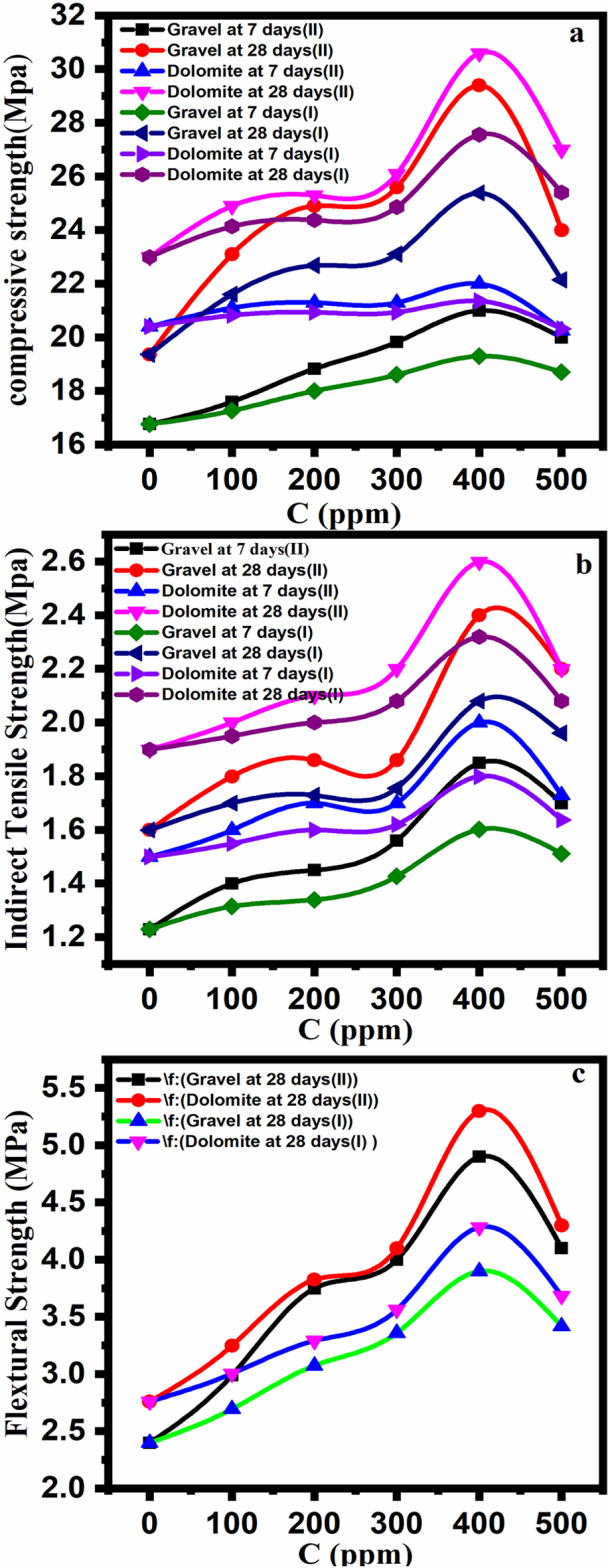
The diagram for the relationship between the concentration of (DHSiMF and DHSiB) and the values of the mechanical properties for concrete.

According to the Table 8, the values of compressive strength for concrete made of gravel aggregate (ACSG) at 7 days increased by 16.8, 17.6, 18.8, 19.8, 20.2, 20, and also 16.8, 17.3, 18, 18.6, 19.3, 18.7 (MPa) were spotted for concentrations 0, 100, 200, 300, 400, and 500 ppm from (DHSiMF and DHSiB) respectively. At 28 days, improvements of 19.6, 23.1, 24.9, 25.9, 29.4, 24 and also 19.6, 21.6, 22.8, 23.1, 25.3, and 22.2 (MPa) were observed for concentrations 0, 100, 200, 300, 400, and 500 ppm, from (DHSiMF and DHSiB) respectively. On the other side, the compressive strength for concrete contains on dolomite aggregate (ACSD) values at 7-days increment by 20.4, 21.1, 21.3, 21.3, 22, 20.3, and 20.4, 20.8, 20.9, 21.3, 20.3 (MPa) were dotted for concentrations 0, 100, 200, 300, 400, and 500 ppm from (DHSiMF and DHSiB) respectively. At 28 days, developments of 23, 24.9, 25.3, 26.1, 30.6, 27, and 23, 24.1, 24.4, 24.9, 27.6, 25.4 (MPa) were scattered for concentrations 0, 100, 200, 300, 400, and 500 ppm from (DHSiMF and DHSiB) respectively. As illustrated in Fig. 17, the compressive strength tests for concrete contain on gravel and dolomite aggregate show that the impacts of (DHSiMF and DHSiB) on old-age concrete are much greater than those of early-day concrete. For example, adding 400 ppm of (DHSiMF and DHSiB) to mixed concrete resulted in an increase in compressive strength of 29.4, 25.3, 30.6, and 27.6 (MPa) at 28 days, whereas the same specimens showed an improvement in compressive strength of only 20.3, 19.3, 22, and 21.3 (MPa) at 7 days for concrete containing gravel and dolomite aggregate, respectively. The best (DHSiMF and DHSiB) content for the 28- and 7-days specimen was 400 ppm but at 28 days are better than 7 days. One can observed that the reasons for these improvements for the compressive strength for concrete contain on gravel and dolomite aggregate in mix concrete due to (DHSiMF and DHSiB) is acted like quite increase bond of contact between gravel, dolomite aggregate and cement molecules, and prevent occur cracks in the concrete. On the other hand, the presence of (DHSiMF and DHSiB) in concrete promotes the formation of micro-crystals in the micro-voids of calcium silicate hydrate gel. Consequently, compressive strength increased^89,90^.

**Table 8:** Compressive strength, indirect tensile strength, and flexure strength for gravel and demode concrete at different concentrations of inhibitor compounds.

		DHSiMF						DHSiB					
		0	100	200	300	400	500	0	100	200	300	400	500
ACSG (Mpa)	7 days	16.8	17.6	18.8	19.8	20	20.2	16.8	17.3	18	18.6	19.3	18.7
	28 days	19.4	23.1	24.9	25.6	29.4	24	19.4	21.6	22.7	23.1	25.3	22.2
ACSD (Mpa)	7 days	20.4	21.1	21.3	21.3	22	20.3	20.4	20.8	20.9	20.9	21.3	20.3
	28 days	23	24.9	25.3	26.1	30.6	27	23	24.1	24.4	24.9	27.6	25.4
AITSG (Mpa)	7 days	1.23	1.4	1.45	1.56	1.85	1.7	1.23	1.3	1.3	1.4	1.6	1.5
	28 days	1.6	1.8	1.86	1.86	2.4	2.2	1.6	1.7	1.73	1.8	2.1	1.96
AITSD (Mpa)	7 days	1.5	1.6	1.7	1.7	2.0	1.73	1.5	1.55	1.6	1.62	1.8	1.7
	28 days	1.9	2.0	2.1	2.2	2.6	2.2	1.9	1.95	2	2.1	2.32	2.1
AFSG (Mpa)	28 days	2.4	2.99	3.75	4	4.9	4.1	2.4	2.7	3.1	3.4	3.9	3.4
AFSD (Mpa)	28 days	2.76	3.25	3.83	4.1	5.3	4.3	2.76	3	3.3	3.6	4.3	3.7

### Tensile strength

The results of the tensile strength test for concrete of the 7 and 28 days are shown in Table 8 and Fig. 17. The results show that (DHSiMF and DHSiB) increase the indirect tensile strength of concrete by a large amount. The concrete tensile strength test, the concrete is made of gravel and dolomite aggregate, and has a strength of up to 1.8, 2.3, 2.4, 2.6, 1.6, 1.8, 2.1, and 2.32 MPa, which includes 400 ppm from (DHSiMF and DHSiB) and is higher than that of conventional control concrete at 7 and 28 days. The concrete indirect tensile strength (MPa) curing at 7 and 28 days at the addition of different concentrations of (DHSiMF and DHSiB) is shown in Fig. 10. It has been added at these concentrations of 0, 100, 200, 300, 400, and 500 ppm of (DHSiMF) to the concrete. The tensile strength of the concrete samples made of gravel and dolomite that were immersed for 28 days and 7 days increased by 1.6–2.2, 1.9–2.2, 1.3–1.7, 1.5–1.73, and 1.9–2.2, respectively. In other words, the indirect tensile strength values for concrete containing gravel and dolomite aggregates when adding the same concentration of (DHSiB) to the concrete at 28-days and 7-days were reinforced by 1.7–1.96, 1.95–2.1, 1.3–1.5, and 1.53–1.7 (MPa), respectively. Overall, the results indicate that the chemical interaction between (DHSiMF and DHSiB) and the concrete will increase in the indirect tensile strength of the concrete made from gravel and dolomite aggregate. The effect of the addition (DHSiMF and DHSiB) to the concrete on the indirect tensile strength, such as compressive strength, when the concrete is immersed at 28-days-old was quite greater than those immersed at 7-days-old. For both 7-day and 28-day immersed concretes, 400 ppm is the best amount of (DHSiMF and DHSiB) to get the best tensile strength. Just like with compressive strength, the tensile strength of concrete keeps going down at any concentration of DHSiMF and DHSiB more than 400 ppm is added^91^.

### Flexural strength

The results for the flexural strength test of concrete, which includes different concentrations of DHSiMF and DHSiB, which were immersed for 7 and 28 days, are shown in Table 8 and Fig. 17. The flexural strength of the concrete improved when (DHSiMF and DHSiB) were added to the concrete samples. Flexural strength is one of the mechanical properties of concrete. When adding 400 ppm of (DHSiMF and DHSiB) to the control concrete sample, the flexural strength reached to the maximum increased. The values of flexural strengths of up to 34, 47.2, 35, and 28% for concrete made of gravel and dolomite, respectively. The flexural strengths of concrete made of gravel and dolomite are higher than those of ordinary concrete. Figure 17 shows how the improvement of the flexural strength of concrete with the concentration’s changes (DHSiMF and DHSiB). Different concentrations of (DHSiMF and DHSiB) added to gravel and dolomite aggregate concrete improved flexural strength by 20–42%, 11–29%, 15–35%, and 7–24%, respectively, at 28 days. The 400 ppm concentration of (DHSiMF and DHSiB) is a good concentration which leads to the improvement in the flexural strength of the concrete, shown in Fig. 10. This concentration works well with the concrete specimen that is immersed in a 28-day-old. Furthermore, the final result, the 500 ppm concentration of (DHSiMF and DHSiB), was not the optimal content for improving the compressive strength, indirect tensile strength, and flexural strength of concrete made of gravel and dolomite aggregate at 28 days, despite the fact that the improvement in the compressive strength, indirect tensile strength, and flexural strength was still greater than that of concrete made of gravel and dolomite aggregate without (DHSiMF and DHSiB). This happens because DHSiMF and DHSiB have such a large specific surface area that they give off a lot of surface energy^92^.

Improving the mechanical properties of reinforcement Concrete requires a good curing process, and the curing process is carried out in several ways: water curing, membrane curing, and steam curing. Water curing reduces water loss from the concrete surface by maintaining continuous wetting of the concrete’s exposed surface. This is accomplished by spraying or sprinkling water or curing ingredients over the concrete surface in order to maintain a continuously moist surface. Moisture in the body of the concrete is prevented from evaporating, thereby strengthening the concrete. Ponding, sprinkling, fogging, mist curing, and wet covers are water curing techniques^93,94^. The process of curing by water spray leads to the exposure of the concrete reinforcement rebar to an aqueous medium, which makes it susceptible to corrosion.

**Figure 18:**
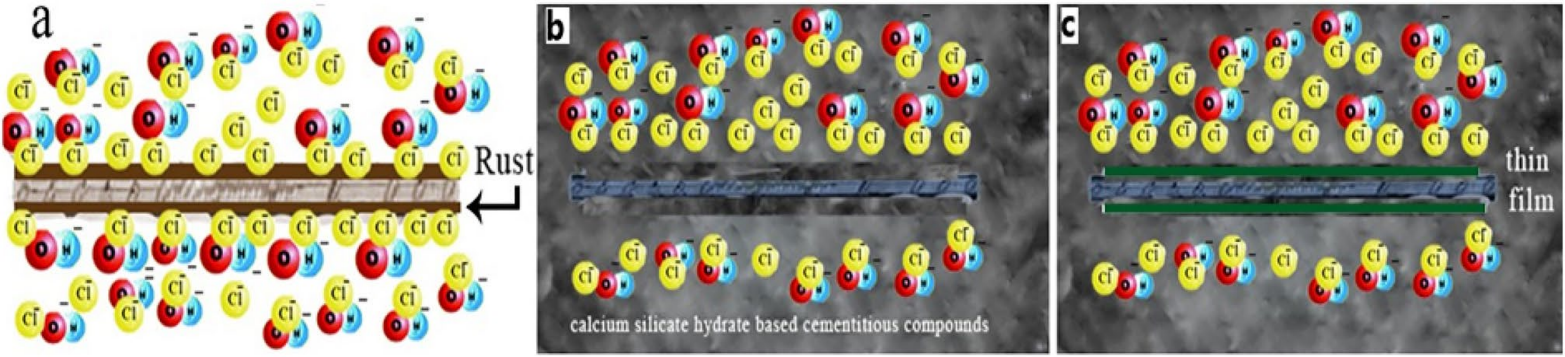
The corrosion reaction mechanism (**a**) without inhibitor (**b**) concrete mixed without inhibitor (**c**) concrete mixed with inhibitor.

Two inhibitor materials for corrosion (DHSiMF and DHSiB) were added to the concrete mix in which the rebar is protected from corrosion. Figure 18 shows the effect of concrete without and with corrosion inhibitors on protecting rebar from corrosion. Figure 18a shows the effect of chlorine and oxygen ions on the formation of the rust layer on the surface of the rebar without concrete mixed and inhibitor materials. Figure 18b shows the layer formation as calcium silicate hydrate-based cementitious compounds from concrete mixed on top of the rebar. These layers behave like cotton layers that surround the reinforcing rebar, which increases its protection from the influence of the aqueous medium. On the other hand, Fig. 18c shows that adding the inhibitor compounds to concrete during the concrete mixing and casting led to film formation adsorbed under the cotton layer, which is attached to the rebar surface. The rebar has been protected from corrosion by these layers.

### Mechanism of corrosion inhibition

**Figure 19:**
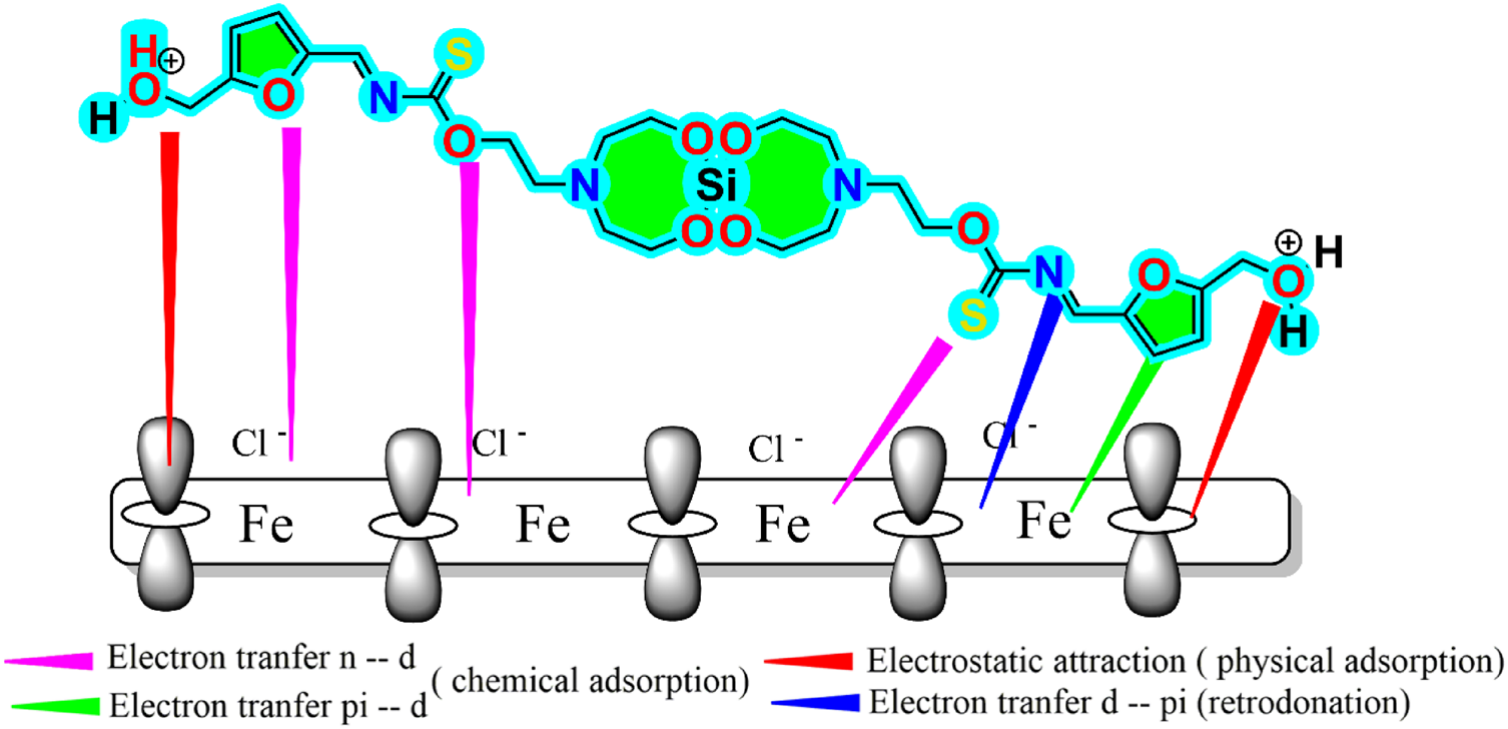
The inhibitive adsorption mechanism of DHSiMF for steel corrosion in HCl-containing environment.

The schematic design in Fig. 19 may be used to understand how DHSiMF and DHSiB prevent steel corrosion. Aggressive species (Cl^–^ and H_3_O^+^) may easily access the steel surface in the blank media, causing significant corrosion of the metal. The cathodic process involves the reduction of H^+^ ions in the HCl-containing aqueous environment, whereas the anodic process involves the dissolving of steel ions. Anodic reaction mechanism without inhibitor:

$$Fe+{H}_{2}O\leftrightarrow Fe{({H}_{2}O)}_{ads},$$

$$Fe{({H}_{2}O)}_{ads}+{Cl}^{-}\leftrightarrow Fe{{(Cl}^{-})}_{ads}+{H}_{2}O,$$

$$Fe{{(Cl}^{-})}_{ads}+{OH}^{-}\to Fe{OH}^{+}+{Cl}^{-}+2e,$$

$$Fe{OH}^{+}+{H}^{+}\leftrightarrow {Fe}^{2+}+{H}_{2}O,$$

Cathodic reaction mechanism without inhibitor:

$$2{H}^{+}+{2e}^{-}\to {H}_{2}.$$

After the addition of Schiff base derivatives into the HCl solution, DHSiMF and DHSiB could adsorb on steel surface by mixed chemisorption and physisorption as indicated from experimental ΔG values. The adsorbed H_2_O, H3O^+^ and Cl^-^ molecules on the steel surface may be replaced by molecules of DHSiMF and DHSiB.

Anodic reaction mechanism after adding DHSiMF and DHSiB inhibitor:

$$Fe{({H}_{2}O)}_{ads}+\mathrm{DHSiMF}\leftrightarrow Fe{(\mathrm{DHSiMF}}_{ads})+{H}_{2}O,$$

$$Fe{{Cl}^{-}}_{ads}+\mathrm{DHSiMF}\leftrightarrow Fe {Cl}^{-}\dots \dots \dots \dots \mathrm{DHSiMF},$$

$$Fe+\mathrm{DHSiMF}\leftrightarrow Fe({\mathrm{DHSiMF})}_{ads},$$

$$Fe({\mathrm{DHSiMF})}_{ads}\leftrightarrow {Fe}^{2+}+\mathrm{DHSiMF}+{ne}^{-},$$

$${\mathrm{DHSiMF}}_{aq}+{{H}_{2}O}_{ads}\leftrightarrow {\mathrm{DHSiMF}}_{ads}+{{H}_{2}O}_{aq}.$$

According to Molecular simulations, DHSiMF and DHSiB could progressively pass through the aqueous layer and adsorb on the surface of steel in a pattern that was virtually parallel. This was made possible through several active adsorption sites like hetero atoms, unsaturated bonds and aromatic moieties adhering to the steel surface. The protonated parts in DHSiMF and DHSiB molecules can be adsorbed on the steel surface via electrostatic interaction with a little amount of Cl^-^ in acidic solution, which is attributed to physical adsorption. Also, the unprotonated reactive adsorption sites like the heteroatoms and π electrons will then interact with the vacant d-orbitals of Fe to form coordination bonds which is attributed to chemical adsorption. Additionally, a certain quantity of electrons accumulates on the surface of carbon steel due to the sharing of electrons from the DHSiMF and DHSiB with the vacant d-orbitals of Fe. This additional negative charge on the surface of carbon steel has to be discharged by retro-donation from the unoccupied d-orbitals of Fe to a vacant * (antibonding) orbital in the DHSiMF and DHSiB molecules. This improves DHSiMF and DHSiB absorption and gives the carbon surface better corrosion protective capabilities.

Also, Table 9 compares the examined Schiff bases with other inhibitors of steel in acidic media that have been reported. The comparative table makes it evident that the compounds we evaluated had higher efficiency and stronger protective capabilities.

**Table 9:** Comparison of the inhibition efficiencies (IE %) of surfactant with other inhibitors from literature.

Inhibitor	Medium	Substrate	Concentration	IE%	Ref
	5% HCl	steel	5 × 10^−3^ mol/dm^3^	PDP:90.9%, EIS: 90%	^18^
	1 M HCl	Mild steel	250 mg L^−1^	PDP:92.8%, EIS: 91%	^44^
	1 M H_2_SO_4_	C-steel	10^−2^ M	PDP: 79%, EIS: 66.5%	^47^
	1 M HCl	C-steel	10^−2^ M	PDP: 98%, EIS: 97%	This study

## Conclusions

Two novel silicon-based Schiff-base compounds, (DHSiMF) and (DHSiB), were evaluated as corrosion inhibitors for carbon-steel corrosion by using electrochemical and weight loss methods. The obtained efficiency results for both inhibitors are excellent in an aqueous medium of 1 M HCl. The inhibition efficiency of DHSiMF of carbon steel is greater than DHSiB. According to ΔE_corr_ values from the extrapolation of the Tafel curve, the DHSiMF and DHSiB may be categorized as mixed-type and anodic inhibitors, respectively. The isotherm of Langmuir has provided the best overview of the adsorption on carbon steel. The values of ∆Goads for DHSiMF and DHSiB at temperatures (ranging from 303 to 333 K) are between-34.42 kJ mol^–1^ and − 37.51 kJ mol^–1^, so Physical and chemical adsorption is therefore considered to be the best explanation for the adsorption process. It was also noted that ($\Delta {H}_{a}^{*}$) values for DHSiMF are positive and refer to the endothermic nature of the system, while the negative values of ($\Delta {H}_{a}^{*}$) for DHSiB refer to the exothermic nature of the metal oxidation process. On the other hand, the best (DHSiMF and DHSiB) content for the 28 and 7-day specimens was 400 ppm, but at 28 days, the mechanical properties for the concrete mix were better. One can observe that the reasons for these improvements in the compressive strength of concrete containing gravel and dolomite aggregate in mixed concrete are that (DHSiMF and DHSiB) act as a quite increased bond of contact between gravel, dolomite aggregate, and cement molecules, preventing the occurrence of cracks in the concrete. On the other hands, the presence of (DHSiMF and DHSiB) in concrete encourages the formation of microcrystals in the micro-voids of calcium silicate hydrate gel. The compressive strength has increased as a result.

## Data Availability

The datasets used and/or analysed during the current study is available from the corresponding author on reasonable request.
