# Author Correction: Synthesis and applications of novel Schiff base derivatives as corrosion inhibitors and additives for improvement of reinforced concrete

**DOI:** 10.1038/s41598-024-61705-z

**Published:** 2024-05-16

**Authors:** Ehab S. Gad, Mohamed A. Abbas, Mahmoud A. Bedair, Olfat E. El-Azabawy, Shymaa M. Mukhtar

**Affiliations:** 1 Chemistry Department, College of Science and Arts, Jouf University, Alqurayat, Saudi Arabia; 2 Egyptian Petroleum Research Institute (EPRI), Cairo, 11727 Egypt; 3 Department of Chemistry College of Science and Arts, University of Bisha, P.O. Box 101, 61977 Al-Namas, Saudi Arabia; 4 Civil Engineering Department, Higher Technological Institute, 10th of Ramadan City, Sharqeya Egypt

Correction to: *Scientific Reports* 10.1038/s41598-023-41165-7, published online 12 September 2023

The original version of this Article contained errors in the Affiliations.

In the original version of this Article Ehab S. Gad was incorrectly affiliated with ‘Department of Chemistry, Faculty of Science (Men’s Campus), Al-Azhar University, Nasr City 11884, Cairo, Egypt’. The correct affiliation is listed below.

Chemistry Department, College of Science and Arts, Jouf University, Alqurayat, Saudi Arabia

In addition, the affiliation ‘Department of Chemistry, Faculty of Science (Men’s Campus), Al-Azhar University, Nasr City 11884, Cairo, Egypt’ was removed.

As a result, the Affiliations have been renumbered.

The original Article has been corrected.

